# Specificity of RNAi, LNA and CRISPRi as loss-of-function methods in transcriptional analysis

**DOI:** 10.1093/nar/gky437

**Published:** 2018-06-01

**Authors:** Lovorka Stojic, Aaron T L Lun, Jasmin Mangei, Patrice Mascalchi, Valentina Quarantotti, Alexis R Barr, Chris Bakal, John C Marioni, Fanni Gergely, Duncan T Odom

**Affiliations:** 1 Cancer Research UK Cambridge Institute, University of Cambridge, Li Ka Shing Centre, Robinson Way, Cambridge CB2 0RE, UK; 2 Institute of Cancer Research, 237 Fulham Road London SW3 6JB, UK; 3 European Bioinformatics Institute, European Molecular Biology Laboratory (EMBL-EBI), Wellcome Genome Campus, Hinxton, Cambridgeshire CB10 1SD, UK; 4 Wellcome Trust Sanger Institute, Wellcome Genome Campus, Hinxton, Cambridgeshire CB10 1SA, UK

## Abstract

Loss-of-function (LOF) methods such as RNA interference (RNAi), antisense oligonucleotides or CRISPR-based genome editing provide unparalleled power for studying the biological function of genes of interest. However, a major concern is non-specific targeting, which involves depletion of transcripts other than those intended. Little work has been performed to characterize the off-target effects of these common LOF methods at the whole-transcriptome level. Here, we experimentally compared the non-specific activity of RNAi, antisense oligonucleotides and CRISPR interference (CRISPRi). All three methods yielded non-negligible off-target effects in gene expression, with CRISPRi also exhibiting strong clonal effects. As an illustrative example, we evaluated the performance of each method for determining the role of an uncharacterized long noncoding RNA (lncRNA). Several LOF methods successfully depleted the candidate lncRNA but yielded different sets of differentially expressed genes as well as a different cellular phenotype upon depletion. Similar discrepancies between methods were observed with a protein-coding gene (*Ch-TOG/CKAP5*) and another lncRNA (*MALAT1*). We suggest that the differences between methods arise due to method-specific off-target effects and provide guidelines for mitigating such effects in functional studies. Our recommendations provide a framework with which off-target effects can be managed to improve functional characterization of genes of interest.

## INTRODUCTION

The ability to specifically reduce the expression of a particular gene is fundamental for establishing its loss-of-function (LOF) phenotype in cells and organisms, and is frequently the only way to infer gene function. The most commonly used LOF methods are RNA interference (RNAi), antisense oligonucleotides and CRISPR interference (CRISPRi) (1). RNAi uses small interfering RNA molecules to deplete target transcripts by triggering their degradation (2). Efficient depletion of genes can also be achieved with antisense oligonucleotides (3); the most widely used antisense approach involves locked nucleic acids (LNAs) (4), where the presence of the LNA modification within an RNA:DNA hybrid triggers RNase-H-mediated degradation of the target transcript in the nucleus (5) or even depletion of the nascent transcript (6). Most recently, the CRISPR/Cas9 system has been adapted to inhibit the expression of single genetic locus. Deactivated Cas9 (dCas9) fused to a Krüppel-associated box (KRAB) repression domain can be directed to a specific genomic locus to prevent transcription, an approach known as CRISPR interference (CRISPRi) (7–10). This allows repression of a targeted locus without editing the genome, thus avoiding unintentional deletion of active regulatory elements (11).

These three approaches have been successfully applied in the literature to characterize the function of a variety of coding and non-coding genes in a range of biological systems (9,12–19). Of increasing interest is the application of LOF methods to deplete long noncoding RNAs (lncRNAs), of which there are tens of thousands in the mammalian genome. Many of these are involved in the regulation of diverse cellular processes and act as key players in chromatin organisation and (post-)transcriptional gene regulation (20,21), but the function of the majority of lncRNAs is unknown. Indeed, the FANTOM6 project aims to systematically elucidate the function of lncRNAs in the human genome using these LOF methods. However, a number of studies in the lncRNA field have shown discrepancies between the cellular and molecular phenotypes obtained with different depletion methods, even in the same cellular background (22–28). This raises into question the reliability of these methods for functional characterization of lncRNAs and their target genes.

When using any LOF method, a critical consideration is whether the expression levels of non-target genes are inadvertently perturbed. These off-target effects arise from non-specific activity of the knockdown technology when exposed to the endogenous pool of total RNAs. For example, RNAi and antisense oligonucleotides with sufficient complementarity to non-target transcripts may cause unintended repression of those genes (29–33). Widespread genome binding and modest off-target effects have also been reported for the dCas9–KRAB system (34,35). However, to the best of our knowledge, little work has been performed in the literature to characterize the off-target activity of each of these commonly used LOF methods at a transcriptome-wide level.

In this study, we comprehensively quantified the off-target effects associated with each LOF strategy, with a particular focus on the transcriptome. Exploiting the HeLa cell line as a powerful and widely used model for LOF studies, we performed RNA sequencing (RNA-seq) with a range of negative controls for each method and used differential expression analyses between control samples to characterize off-target effects. The identities of the genes affected by off-target activity in RNAi and LNA approaches were notably dependent on the sequences of the siRNA/antisense oligonucleotides used. Introducing dCas9–KRAB to a polyclonal population of HeLa cells caused few transcriptional perturbations, with limited sequence-dependent off-target effects. However, single cell cloning from this population resulted in a unique and reproducible transcriptional signature.

To illustrate the impact that differences between methods can have on understanding gene function, we show that depletion of lncRNA with unknown function using different LOF methods can lead to different biological conclusions. We also demonstrate that discrepancies between methods are present in the molecular phenotypes generated upon depletion of a protein-coding gene (*Ch-TOG/CKAP5*) and another lncRNA (*MALAT1*). We suggest that these differences are caused by method-specific off-target effects, and provide recommendations to manage these effects based on our experimental design. This includes the use of multiple negative controls generated from each step of the protocol, and the use of a log-fold change threshold during the differential expression analysis. Our aim is to provide guidelines with which off-target effects can be mitigated to improve functional characterization of genes of interest.

## MATERIALS AND METHODS

### Cell culture

HeLa and HEK293T cells were maintained in Dulbecco's modified Eagle's medium (Sigma Aldrich, D6429) supplemented with 10% Fetal bovine serum (FBS, Thermo Fisher Scientific). Both cell lines were obtained from the American Type Culture Collection (ATCC) and were cultured at 37°C with 5% CO_2_. HeLa Kyoto (EGFP-α-tubulin/ H2B-mCherry) cells were obtained from Jan Ellenberg (EMBL Heidelberg) (36) and cultured in DMEM with 10% FBS (37). All cell lines were verified by short tandem repeat (STR) profiling and tested negative for mycoplasma contamination.

### Single-molecule RNA fluorescent *in situ* hybridization (FISH)

Cells were grown on coverslips, briefly washed with PBS and fixed with PBS/3.7% formaldehyde at room temperature for 10 min. Following fixation, cells were washed twice with PBS. The cells were then permeabilised in 70% ethanol for at least 1 h at 4°C. Stored cells were briefly rehydrated with Wash Buffer (2× SSC, 10% formamide, Biosearch) before FISH. The Stellaris FISH Probes (*SLC25A25-AS1* exonic probes Q570; sequences in Supplementary Table S4) were added to the hybridization buffer (2× SSC, 10% formamide, 10% dextran sulfate, Biosearch) at a final concentration of 250 nM. Hybridization was carried out in a humidified chamber at 37°C overnight. The following day, the cells were washed twice with Wash Buffer (Biosearch) at 37°C for 30 min each. The second wash contained DAPI for nuclear staining (5 ng/ml). The cells were then briefly washed with 2× SSC and then mounted in Vectashield (Vector Laboratories, H-1000). Images were captured using a Nikon TE-2000 inverted microscope with NIS-elements software, a Plan Apochromat 100x objective and an Andor Neo 5.5 sCMOS camera. We acquired 25 optical slices at 0.3 μm intervals. Images were deconvolved with Huygens Professional and projected in two dimensions using ImageJ.

### Plasmids and antibodies

Plasmids used in this study were pHR-SFFV-dCAS9-BFP-KRAB (Addgene, #46911), pU6-sgRNA EF1Alpha-puro-T2A-BFP (Addgene, #60955), second-generation packaging plasmid psPAX2 (Addgene, #12260) and the envelope plasmid pMD2.G (Addgene, #12259). pHIV-Zsgreen (Addgene #18121) and LincExpress-mCherry (modified version of pLenti6.3/TO/V5-DEST, kindly provided by John Rinn, Harvard University) were used as positive controls for transduction efficiency. Cas9 antibody was obtained from Cell Signaling (#14697, dilution 1:1000) and β-tubulin antibody was purchased from Sigma (#T019, dilution 1:2000).

### RNAi- and LNA-mediated gene depletion

HeLa cells were transfected with Lipofectamine RNAiMax reagent (Thermo Fischer Scientific) following the manufacturer's instructions. All experiments to measure the effect of depletion were done 48 hours after transfection. The siRNAs (Thermo Fischer Scientifc) and LNA Gapmers (Exiqon) were used at a final concentration of 50 nM and 25 nm, respectively. siRNA and LNA sequences are listed in Supplementary Tables S5 and S6, respectively.

### Western blotting

Cells were grown in a six-well plate, trypsinized, pelleted and washed twice with PBS. The pellet was lysed in lysis buffer (50 mM Tris–HCl, pH 8, 125 mM NaCl, 1% NP-40, 2 mM EDTA, 1 mM PMSF and protease inhibitor cocktail [Roche]) and incubated on ice for 25 min. Samples were centrifuged for 3 min at 12 000 × g and 4°C. Supernatant was collected and protein concentration was determined using the Direct Detect^®^ Spectrometer (Merck Millipore). Proteins (25 μg) were denatured, reduced, and separated with Bolt® 4–12% Bis–Tris Plus Gel (Thermo Fisher Scientific) in MOPS buffer (Thermo Fisher Scientific, B0001-02). Proteins were then transferred to nitrocellulose membrane and blocked with 5% nonfat milk in TBS-T (50 mM Tris, 150 mM NaCl, 0.1% Tween-20) for 1 h at room temperature. Membranes were incubated with primary antibodies in 5% milk in TBS-T. After overnight incubation at 4°C, the membranes were washed with TBS-T and incubated with HRP secondary antibodies (GE Healthcare Life Sciences, 1:5000), and immunobands were detected with a Pierce ECL Western Blotting Substrate Substrate (Thermo Fischer Scientific, 32106). An uncropped scan of the immunoblot (Figure 2) is shown in Supplementary Figure S20.

### Time-lapse microscopy

HeLa cells (10 000 cells) were cultured in 8-well chamber slides (Ibidi) with 200 μl/well of normal HeLa medium (DMEM, 10% FBS). Thirty minutes before live-cell imaging, the medium was replaced with imaging medium (DMEM fluorobrite, A1896701, Thermo Fisher Scientific, supplemented with 10% FBS and 4 mM Glutamax) containing 300 nM SiR–Hoechst (Spirochrome, SC007). SiR–Hoechst was present in the medium throughout imaging (Figure 4E) while no SiR–Hoechst was used for live-cell imaging after depletion of *Ch-TOG* and*MALAT1* (Supplementary Figures S1 and S2). HeLa Kyoto cells were plated in the same way but imaging was performed in DMEM medium with 10% FBS. Time-lapse microscopy was performed for both cell lines 48 h after transfection with RNAi, LNA or CRISPRi transduction. Mitotic duration was measured as the time from nuclear envelope breakdown (NEBD) until anaphase onset, based on visual inspection of the images. Live-cell imaging was performed using a Zeiss Axio Observer Z1 microscope equipped with a PLAPO 0.95NA 40× dry objective (Carl Zeiss Microscopy) fitted with a LED light source (Lumencor) and an Orca Flash 4.0 camera (Hamamatsu). Four positions were placed per well and a z-stack was acquired at each position every 10 min for a total duration of 12 h. Voxel size was 0.325 μm × 0.325 μm × 2.5 μm. Zen software (Zeiss) was used for data collection and analysis. Throughout the experiment, the cells were maintained in a microscope stage incubator at 37°C in a humidified atmosphere of 5% CO_2_.

### CRISPR interference (CRISPRi) and gRNA design

For CRISPRi, we used published sgRNA sequences including two negative control sgRNAs and sgRNAs against the *H19* and*MALAT1* lncRNAs (9). We also designed new sgRNA sequences for depletion of *SLC25A25-AS1, Ch-TOG* and *MALAT1* (three additional guides). Each sequence was 20 nt in length and targeted a genomic window of −50 to +200 bp relative to the transcription start site (TSS) (Supplementary Table S7). The location of the TSS was determined using the NCBI RefSeq database. The MIT CRISPR (http://crispr.mit.edu) and the gUIDEbook™ gRNA design (Desktop Genetics Ltd) web tools were used to design the gRNA sequence. Potential off-target effects were analysed with the MIT CRISPR and the CRISPR RGEN Cas-OFFinder web tools to obtain all genomic sites with no more than 4 mismatches to the input sequence. We used the Basic Local Alignment Search Tool to determine whether any reported sites were located within annotated genes other than the intended target, and if so, the guide sequence was discarded. Additional sequences were added to the sgRNA sequences to obtain compatible sticky ends for cloning the DNA insert into the 5′BstXI-BlpI3′ digested backbone of a pU6-sgRNA EF1Alpha-puro-T2A-BFP expression plasmid. gRNA oligos were phosphorylated, annealed and cloned into pU6-sgRNA EF1Alpha-puro-T2A-BFP expression plasmid. All inserts were verified with Sanger sequencing.

### Lentiviral transduction

To produce lentivirus, 4 × 10^6^ of HEK293T cells were plated in a 10-cm dish one day prior to transduction. On the following day, cells were transfected with 15 μg of DNA, composed of 9 μg of the lentiviral vector DNA containing the transgene, 4 μg of psPAX.2 and 2 μg of pMD2.G in a final transfection volume of 1.5 ml (including 45 ul of Trans-Lt1 transfection reagent, Mirus) using OptiMEM medium (Thermo Fisher Scientific). As a positive control for viral infection and to control for any possible effects of lentiviral delivery, we transduced cells with polybrene (5 μg/ml, Sigma) or with pHIV-Zsgreen and LincExpress-mCherry vectors. The transfection mixture was incubated for 25 min at room temperature. Prior to transfection, old medium was replaced by 14 ml of fresh medium and transfection mix was added dropwise to the cells and incubated for 24 h at 37°C. The following day, old medium was replaced by 7 ml of fresh medium and incubated for another 24 h at 37°C. Viral supernatant was collected 48 and 72 h post transfection, spun down at 1800 × g for 5 min at +4°C, and filtered through a 45 μm filter. Ready-to-use virus was stored at +4°C. For long-term storage, viral supernatant was frozen at −80°C.

### FACS analysis and cell sorting

HeLa cells were transduced with lentivirus containing the pHR-SFFV-dCAS9-BFP-KRAB vector together with polybrene (5 μg/ml, Sigma). Twenty four hours after lentivirus transduction, the medium was replaced and the cells were incubated for another 48 hours. HeLa cells were then sorted for the BFP-expressing cells using the BD FACSAria III cell sorter (CRUK Flow Cytometry Core Facility). The expression of BFP fluorescent proteins was detected using MACSQuant VYB (Miltenyi Biotec) and the data were analysed using the FlowJo v7.1 software. BFP-sorted HeLa cells were used for single cell cloning in 96-well plate (clonal cells) or to create a stable non-clonal cell population.

### CRISPRi-mediated depletion

Three to four days after FACS sorting, dCas9–KRAB transduced cells were plated on 12-well plates and infected with lentivirus containing gRNAs targeting *SLC25A25-AS1, MALAT1, Ch-TOG* or *H19*, or with lentivirus containing one of two negative guide RNAs. Lentivirus was diluted with HeLa medium (1:1 dilution) and cationic polymer polybrene was added to facilitate viral transduction (5 μg/ml, Sigma, H9268). After a 24 h incubation, supernatant was removed and fresh medium was added for another 48 hour incubation before RNA collection to evaluate knockdown of the target gene. Non-transduced cells did not receive virus and were used as a negative control.

### RNA extraction, cDNA and quantitative real-time PCR (qPCR)

RNA (1 μg) was extracted with the RNeasy Kit (QIAGEN, 74106) and treated with DNase I following the manufacturer's instructions (QIAGEN, 79254). The QuantiTect Reverse Transcription Kit (QIAGEN, 205313) was used for cDNA synthesis including an additional step to eliminate genomic DNA contamination. Quantitative real-time PCR (qPCR) was performed on a 7900HT Fast Real-Time PCR System (Applied Biosystems) with Fast SYBR Green Master Mix (Life Technologies). Thermocycling parameters were defined as 95°C for 20 s followed by 40 cycles of 95°C for 1 s and 60°C for 20 s. Two reference genes (*GAPDH* and *RPS18*) were used to normalise expression levels using the 2^−ΔΔCT^ method. Sequences of qPCR primers are provided in Supplementary Table S8.

### Subcellular fractionation

RNA was fractionated as described previously (38,39). Briefly, cells from a 150-mm dish were used to isolate RNA from cytoplasmic, nucleoplasmic and chromatin fractions by TRIZOL extraction (Life Technologies). Expression of target genes in each fraction was analysed by qPCR. Data were normalised to the geometric mean of *GAPDH* and *ACTB* levels in each cellular compartment. *MALAT1* and *RPS18* were used as positive controls for chromatin and cytoplasmic fractions, respectively.

### RNA library preparation, sequencing and analysis

RNA-seq libraries were prepared from HeLa cells using the TruSeq Stranded Total RNA Kit with Ribo-Zero Gold (Illumina, RS-122-2303). We generated four biological replicates of cell populations after RNAi, LNA and CRISPRi-mediated depletion of *SLC25A25-AS1* or *H19* with an equal number of replicates for the corresponding negative controls. Indexed libraries were PCR-amplified and sequenced using 125 bp paired-end reads on an Illumina Hiseq 2500 instrument (CRUK Genomics Core Facility). In addition, two biological replicates were generated for RNAi- and LNA-mediated depletion of *Ch-TOG* and *MALAT1* respectively, while three biological replicates were generated for CRISPRi-mediated depletion of *Ch-TOG* and *MALAT1*. These libraries were sequenced using 150 bp paired-end reads on an Illumina Hiseq 4000 instrument (CRUK Genomics Core Facility). Each library was sequenced to a depth of 20–30 million read pairs. Paired-end reads were aligned to the hg38 build of the human genome (40) and the number of read pairs mapped to the exonic regions of each gene was counted for each library (41). Approximately 80% of read pairs contained one read that was successfully mapped to the human reference genome. On average, 74% of all read pairs in each library were assigned into exonic regions and counted. Any outlier samples with very low depth (resulting from failed library preparation or sequencing) were removed prior to further analysis. Differential gene expression analyses were performed using the voom-limma framework (42), where we tested for differential expression above a log_2_-fold change threshold of 0.5 in pairwise contrasts between groups of samples (43). For each contrast, genes with significant differences in expression between groups were detected at a false discovery rate (FDR) of 5%.

### Determination of noncoding potential of lncRNAs

The Coding-Potential Calculator (CPC) ((44), http://cpc.cbi.pku.edu.cn) and the Coding Potential Assessment Tool (CPAT) ((45), http://lilab.research.bcm.edu/cpat/index.php) were used to determine noncoding potential. LncRNAs with CPC score >1 and CPAT score >0.364 were predicted to have protein-coding capacity. The PhyloCSF score was taken from UCSC (https://github.com/mlin/PhyloCSF/wiki, (46)).

### Statistical analysis

The statistical significance of differences between groups was determined by two-tailed Student's *t*-test in all experiments using GraphPad Prism unless indicated otherwise. *P*-values > 0.05 were considered statistically not significant. The differential expression analysis of the RNA-seq data is described in detail in the Supplementary Methods.

## RESULTS

### LNA and RNAi technologies are associated with non-negligible off-target effects

We first verified that the RNAi and LNA methods were able to deplete both protein-coding genes and lncRNAs. We successfully depleted *Ch-TOG/CKAP5*, a microtubule-associated protein required for mitotic spindle assembly, using a pool of four different siRNAs (Supplementary Figure S1A). We were able to recapitulate the well-established cellular phenotype of mitotic delay upon *Ch-TOG* depletion (Supplementary Figure S1B and C) (47,48). For LNA oligonucleotides, we noted that they have mostly been used to deplete nuclear lncRNAs (16,26,49). Thus, to verify their effectiveness, we used LNA oligonucleotides to efficiently deplete *MALAT1* (Supplementary Figure S2A and B), a highly conserved nuclear lncRNA with roles in regulating gene expression, RNA processing and alternative splicing (50–52). Depletion of *MALAT1* in HeLa cells led to mitotic delay (Supplementary Figure S2C and D) as described previously (26). Thus, both technologies were able to deplete target genes at high efficiency and recapitulate the associated cellular phenotypes.

**Figure 1.**
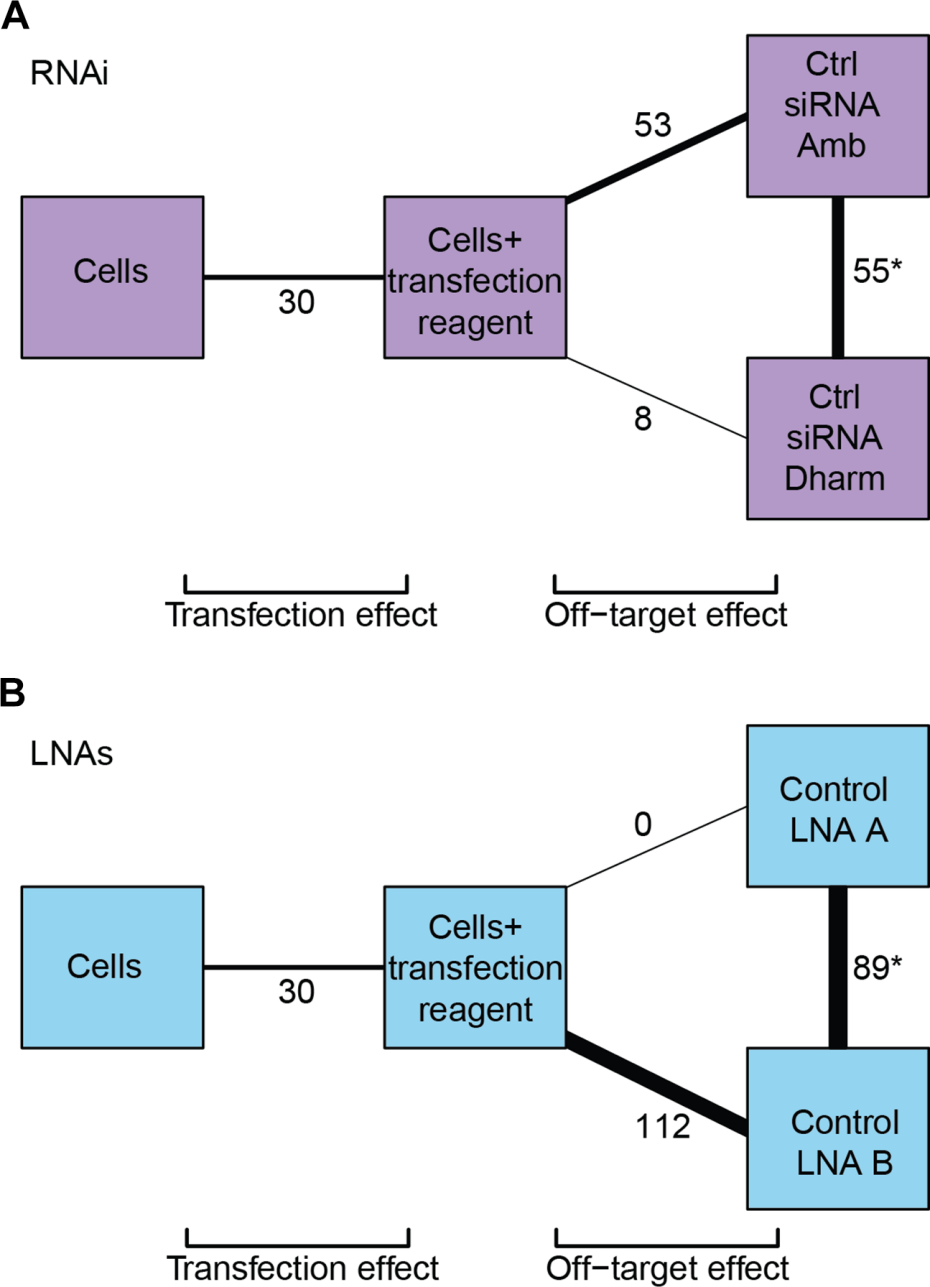
Off-target effects associated with RNAi and LNA oligonucleotides. (**A**) Comparison of the transcriptional differences between untreated cells, cells treated with transfection reagent (RNAiMax) and two negative control siRNAs (Ambion and GE Dharmacon). The number of DEGs between each pair of treatments is labelled and shown as connecting lines of proportional thickness. (**B**) Comparison of the transcriptional differences between untreated cells, cells treated with transfection reagent (RNAiMax) and two negative control LNAs (A and B). The number of DEGs differing between each pair of treatments is labelled as described in A. DEGs for each pairwise comparison were defined at an FDR of 5% testing for a log_2_-fold change significantly greater than 0.5. Lists of DEGs for each comparison are shown in Supplementary Table S1. Note that the comparison between cells treated with transfection reagent and untreated cells is the same between A and B.

We then performed RNA-seq on untreated cells, cells treated with transfection reagent alone, or cells treated with one of two negative control siRNAs (Ambion and GE Dharmacon) (Figure 1A). In principle, treatment with the negative control siRNAs should have no effect on gene expression, as no gene is targeted for depletion. Thus, our experimental design allows us to quantify the transcriptional off-target effects of the two negative control siRNAs, based on the number of differentially expressed genes (DEGs) when compared to cells treated with transfection reagent.

We identified 8 (GE Dharmacon) and 53 genes (Ambion) affected by the introduction of each negative control siRNA at a FDR of 5% (Figure 1A, Supplementary Figure S3), some of which we validated by qPCR (Supplementary Figure S4A). Of these, five genes were affected by both negative control siRNAs, likely representing a general effect of siRNA transfection (Supplementary Figure S4B). The off-target genes affected by either of the negative control siRNAs were not obviously associated with a functional pathway. No single KEGG term contained more than 20% of the DEGs (Supplementary Figure S5A and B, Supplemental Table S1), with most terms containing less than 10%. These results indicate that off-target genes associated with addition of negative control siRNAs do not fall into pathways that would be easy to computationally predict or remove.

We further identified DEGs between cells treated with negative control siRNA from Ambion and those treated with the negative control siRNA from GE Dharmacon. In this comparison, only the siRNA sequence differs between the two controls—thus, any DEGs between the controls must represent sequence-dependent off-target effects, rather than a general effect of siRNA transfection. Comparison between the negative control treatments yielded 55 DEGs, indicating that the perturbations due to non-specific targeting are dependent on the exact sequence of the siRNA used to treat the cells. Again, no common function for this set of DEGs was detectable by KEGG pathway analysis (Supplementary Figure S5C, Supplemental Table S1), with fewer than 10% of DEGs associated with any KEGG term.

We also generated transcriptomic profiles for negative controls at each step of the depletion protocol using LNA Gapmers (Exiqon). Treating cells only with transfection reagent led to perturbation of 30 genes (Figure 1B, Supplementary Figure S6) compared to untreated cells. Treatment using either of two different negative control LNAs yielded zero (control LNA A, Exiqon part number 300611-00) and 112 (control LNA B, Exiqon part number 300615-00) DEGs compared to the transfection control, some of which we validated by qPCR (Supplementary Figure S7A). Comparison between the two negative control LNAs identified 89 DEGs. Applying the same reasoning as described above for RNAi, these 89 genes represent the sequence-dependent off-target effects of the LNA approach. Similar to RNAi, KEGG pathway analysis of off-target effects using negative LNA controls did not reveal any common function for the DEGs (Supplementary Figure S7B and C; Supplementary Table S1).

We note that our DE analyses used a log-fold change threshold of 0.5 based on the TREAT method (43). This prioritizes the detection of genes with large log-fold changes in expression that are more likely to perturb the biology of the cell. For comparison, we repeated our analysis without any log-fold change threshold, which resulted in over an order of magnitude increase in the number of DEGs in most comparisons between negative controls (Supplementary Figure S8A and B). This motivates the use of a threshold in our DE analysis, to avoid having to consider a much larger number of genes with small log-fold changes that are only weakly affected by off-target activity.

In summary, a non-negligible number of genes are affected by off-target activity with both RNAi and LNA technologies. The sequence-dependent nature of the off-target effects has important implications for how these methods can be used to study transcriptional regulation. In particular, it strongly suggests that generic negative controls cannot accurately recapitulate non-specific changes in expression that arise when targeting a particular gene.

### CRISPRi with single cell cloning introduces transcriptional variation

**Figure 2.**
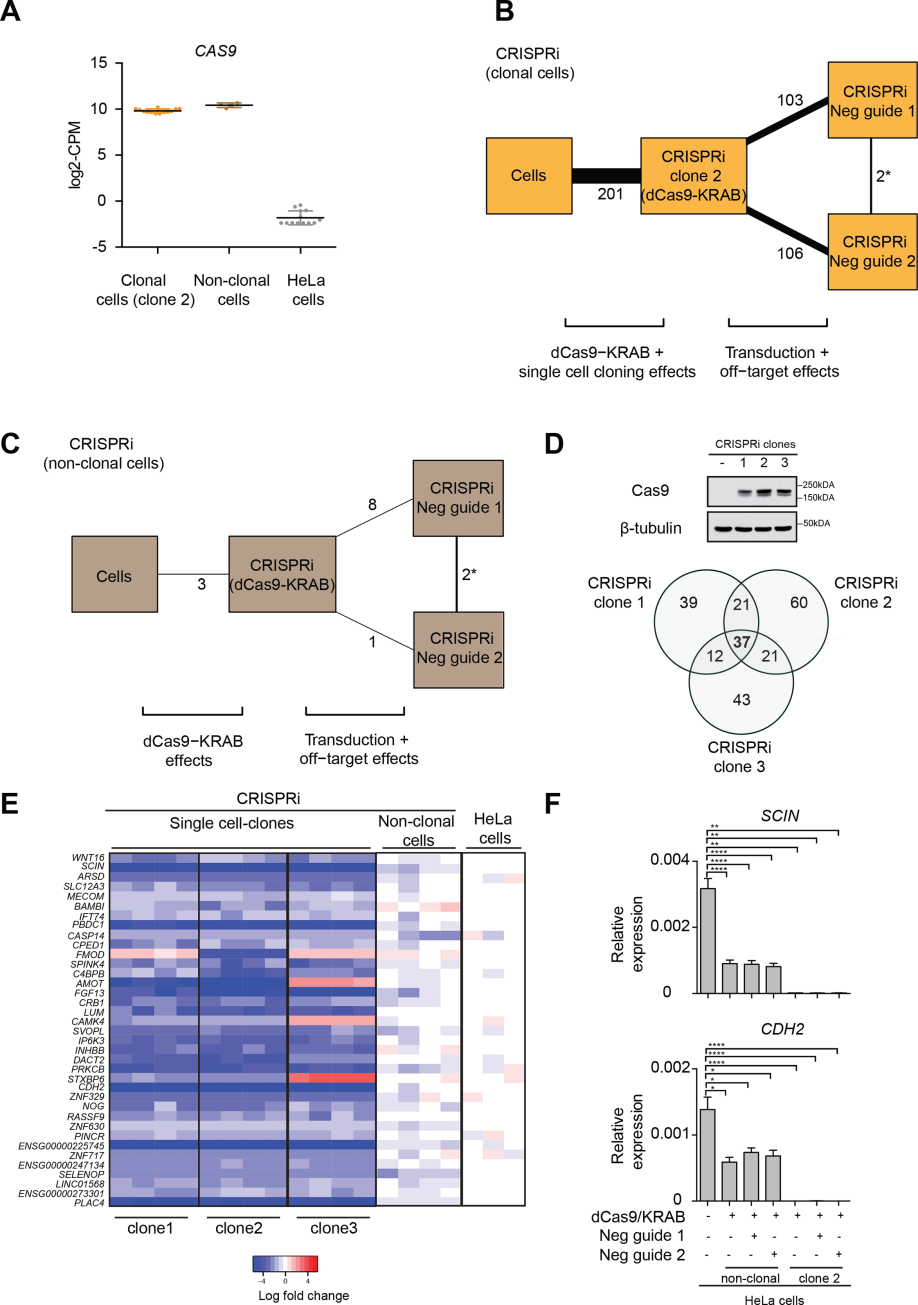
Clonal variations in CRISPRi and their associated off-target transcriptional effects. (**A**) Expression in counts-per-million (CPM) of *Cas9* in CRISPRi clonal, CRISPRi non-clonal and untransduced HeLa cells. Clone 2 was used for showing *Cas9* expression in CRISPRi clonal cells. (**B)** Comparison of the transcriptional differences between parental HeLa untreated cells, CRISPRi clones expressing only dCas9–KRAB (clone 2) and clones treated with two negative guide RNAs (negative guide 1 and 2). The number of genes differing between each pair of treatments is labelled and shown as connecting lines of proportional thickness. (**C**) Comparison of the transcriptional differences between parental HeLa untreated cells, non-clonal CRISPRi cells expressing dCas9–KRAB and non-clonal cells treated with two negative guide RNAs (negative guide 1 and 2). The number of genes differing between each pair of treatments is labelled as described in B. (**D**) Expression of dCas9–KRAB in three different CRISPRi clones derived from single cell cloning, confirmed by immunoblot using a Cas9 antibody. β-tubulin was used as a loading control. A Venn diagram of DEGs detected in the three different clones against untransduced cells in the absence of any guide RNAs identified 37 genes as a common transcriptional signature of cloning. The total number of genes in this analysis was 18224 and DEGs were detected at a FDR of 5% with a log_2_-fold change threshold of 0.5. (**E**) Heat map of DEGs from three different CRISPRi clones compared to non-clonal cells and parental untransduced HeLa cells in the absence of any guide RNAs. 33 out of 37 genes were downregulated in clonal cells compared to the parental population. (**F**) Downregulation of two randomly selected DEGs from E (*SCIN* and *CDH2*) was validated by qPCR in clonal cells (clone 2) and in non-clonal populations. Expression levels were normalized to the geometric mean of *GAPDH* and *RPS18*. Error bars, s.e.m. (*n* = 4 biological replicates). Statistical significance by two-tailed Student's *t*-test: *P< 0.05, **P< 0.01, ***P< 0.001 and ****P< 0.0001. Lists of DEGs for each pairwise comparison in B and C are provided in Supplementary Table S1.

CRISPRi-mediated transcriptional inhibition can target gene expression using both non-clonal (9,10,13,14) and single cell derived clonal populations (14,53). To directly compare CRISPRi with other LOF methods, we generated HeLa cells expressing dCas9–KRAB using lentiviral transduction, and confirmed the expression of dCas9–KRAB in both clonally isolated populations of cells and non-clonally isolated populations (Figure 2A). We verified that CRISPRi was effective at repressing expression of *Ch-TOG* (Supplementary Figure S9A) and recapitulated the mitotic phenotype (Supplementary Figure S9B and C), albeit with greater heterogeneity than the phenotype observed with RNAi.

We then transduced one CRISPRi clone (CRISPRi clone 2) and a non-clonal cell population with two negative control guide RNAs (negative control guide 1 or 2). We performed RNA-seq to quantify the off-target effects by comparing the transcriptional profiles before and after treatment with each of the negative control guides. The addition of the negative guide RNAs had large effects in the clonal CRISPRi cells (103 and 106 genes in Figure 2B, Supplementary Figure S10) and minor effects in the non-clonal CRISPRi cells (1 and 8 genes in Figure 2C, Supplementary Figure S11). However, only two genes were differentially expressed between the negative guide RNAs in each CRISPRi strategy. This indicates that the sequence-dependent off-target effects of CRISPRi are minor, consistent with previous studies suggesting that CRISPRi-mediated gene repression is highly specific (8). Thus, comparisons between cells treated with different guide RNAs (negative control or targeting a specific gene) can be safely performed.

Single cell cloning after introduction of dCas9–KRAB provides another potential source of off-target effects. We found 201 DE genes between the parental HeLa cells and clonally-derived cells expressing dCas9–KRAB in the absence of a guide RNA (Figure 2B). To investigate whether the single cell dilution protocol for obtaining dCas9–KRAB-expressing clones could be responsible for the changes in genes expression, we profiled additional CRISPRi clones expressing different levels of dCas9–KRAB (clones 1 and 3; Figure 2D) along with additional independent replicates of CRISPRi clone 2. We detected a core set of 37 DE genes that were consistently differentially expressed in all three clones compared to non-transduced control cells (Figure 2D and E). We did not observe any common pathway for these 37 genes using KEGG analyses (Supplementary Figure S12, Supplementary Table S1) and found no relationship between these genes and their chromosomal location (Supplementary Figure S13). Most of the genes (33 out of 37) were downregulated in all clones, suggesting that they could be direct targets of the repressive KRAB domain. We validated the repression of two of these 37 genes (*SCIN* and *CDH2*) by qPCR (Figure 2F). Importantly, these changes in gene expression are not caused by lentiviral transduction (Supplementary Figure S14). Rather, they are caused by single cell cloning, given that the corresponding comparison between parental HeLa cells and non-clonal cells in the absence of any guide RNA yielded only 3 DEGs (Figure 2C, Supplementary Figure S15C). Thus, stable expression of dCas9–KRAB causes marked changes in the transcriptomic background prior to the depletion of gene of interest.

Widespread genome binding and modest off-target effects have been reported for the dCas9–KRAB system (34,35,54). Therefore, we compared the published binding sites for dCas9 in HEK293T cells (55) with the gene bodies for our core set of 37 differentially expressed genes (Figure 2E) and found no overlap. We performed a similar analysis using the genome-wide mapping of dCas9–KRAB in K562 cells (35) and found only two dCas9–KRAB binding events in these 37 genes. We also found no overlap between our core set of genes with those reported as being differentially expressed upon dCas9–KRAB transduction (35). These poor overlaps may reflect the potential dependency of CRISPRi off-target effects on the cell type, the epigenetic landscape, or other factors (56). More generally, our results suggest that a common blacklist for *in silico* removal of likely affected genes is unlikely to be effective. The absence of a consistent set of genes across three independent studies that have attempted to identify common CRISPRi off-targets suggests that purely computational approaches may not be able to account for off-target effects *a priori*.

### A case study in using LOF methods to deplete a nuclear long noncoding RNA

We applied these three LOF methods—RNAi, LNA oligonucleotides and CRISPRi—to study the regulatory function of a previously uncharacterized lncRNA in HeLa cells. This represents a common use of LOF strategies, given that tens of thousands of uncharacterized lncRNAs exist in the mammalian genome (57,58). Previous studies have successfully depleted lncRNAs using RNAi (12,15,38,59–62), LNAs (6,12,16,17,26,61) and CRISPRi (6,9,12,14,61,63) to determine their functions.

**Figure 3.**
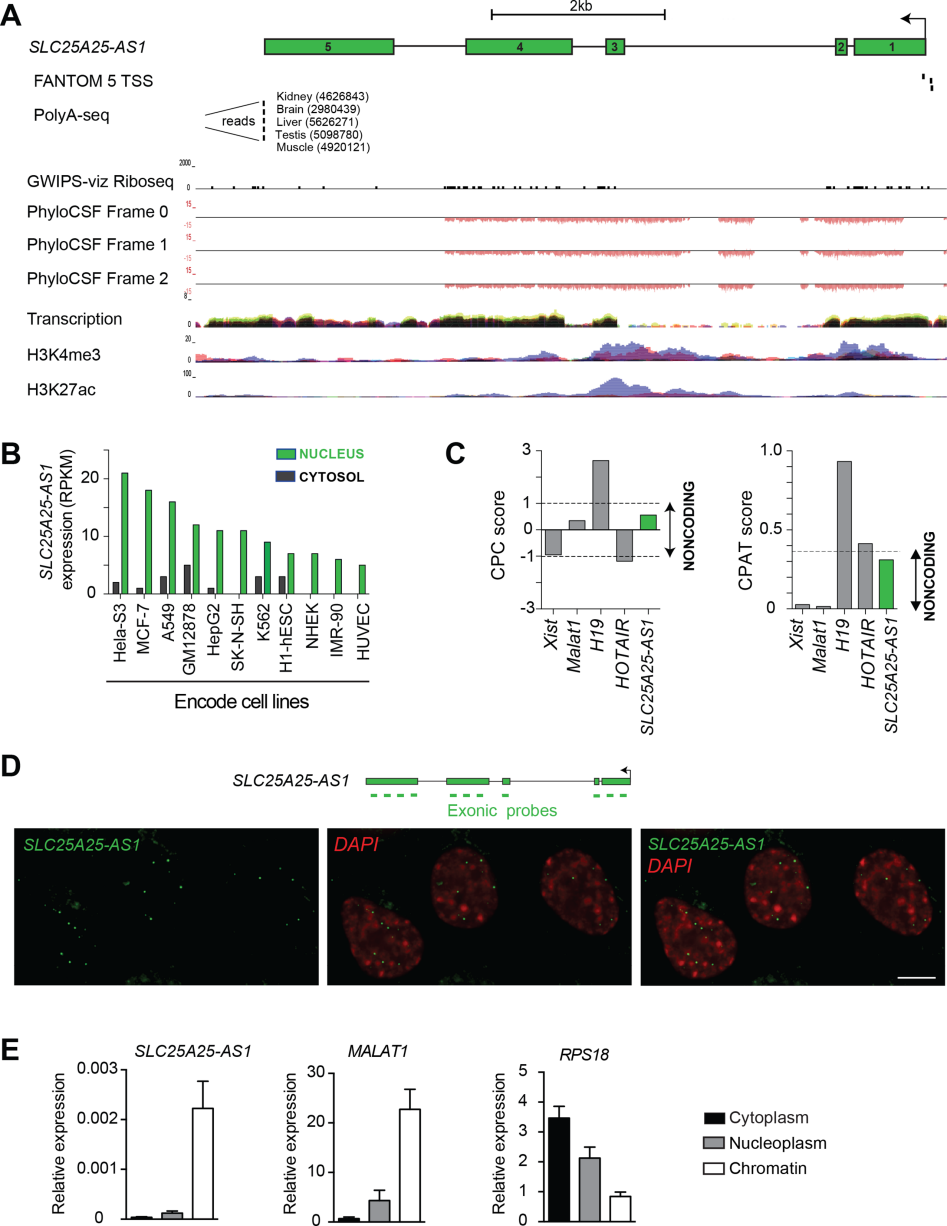
*SLC25A25-AS1* is an archetypal lncRNA expressed in the nucleus. (**A**) Schematic representation of the genomic landscape surrounding *SLC25A25-AS1* (annotated in RefSeq as *loc100289019;* chr9:128108581-128118693, hg38), including three transcriptional start sites (101) and a polyadenylation site (102)*. SLC25A25-AS1* is not occupied by ribosomes (64), shows no protein coding potential (PhyloCSF, (46)), and has clear hallmarks of active transcription in HeLa cells (H3K4me3 and H3K27ac data sets obtained from ENCODE via the UCSC browser). The arrows denote the direction of transcription, and green boxes represent the five exons. Note that all PhyloCSF scores at this locus are negative. (**B**) Expression of *SLC25A25-AS1* in cytosol and nuclei of ENCODE cell lines (www.ebi.ac.uk/gxa/home), shown as reads per kilobase of exon per million reads mapped (RPKM). (**C**) Computational analysis of the mature *SLC25A25-AS1* transcript using the CPC and CPAT tools reveals *SLC25A25-AS1* has low coding potential. (**D**) Nuclear localization of *SLC25A25-AS1* in HeLa cells was determined using single-molecule RNA FISH with exonic probes (green). Nuclei were stained with 4,6-diamidino-2-phenylindole (DAPI). Scale bar represents 5 μm. (**E**) *SLC25A25-AS1* is enriched in chromatin of HeLa cells. RNA distribution in the cytoplasm, nucleoplasm and chromatin was quantified by qPCR, and *RPS18* and *MALAT1* were used as positive controls for the cytoplasmic and chromatin fraction, respectively. Error bars represent the standard error of the mean (s.e.m) values of four independent experiments.

We selected a prototypical lncRNA following published guidelines (11) with specific characteristics, including: (i) previously uncharacterized, (ii) consistently expressed at more than one molecule per cell, (iii) low coding potential, (iv) chromatin hallmarks of active transcription and (v) nuclear localization. Using these criteria, we chose *SLC25A25-AS1* (also known as *loc100289019*) as a spliced and intragenic lncRNA with three promoters and a single 3′ polyadenylation site (Figure 3A). Among all ENCODE cell lines, *SLC25A25-AS1* was most highly expressed in the nucleus of HeLa cells (Figure 3B). We confirmed that *SLC25A25-AS1* has low protein coding potential using PhyloCSF, Coding-Potential Calculator (CPC) and Coding-Potential Assessment Tool (CPAT) (Figure 3C). Furthermore, computational analysis of previously published ribosomal occupancy data indicated no translation of the *SLC25A25-AS1* transcript in HeLa cells (64) (Figure 3A, Riboseq track). Nevertheless, the genomic locus was actively transcribed based on the presence of both histone H3 lysine 4 trimethylated (H3K4me3) and histone H3 lysine 27 acetylated (H3K27ac) histones at the predicted *SLC25A25-AS1* locus (Figure 3A). We experimentally confirmed nuclear localization of *SLC25A25-AS1* by single-molecule RNA FISH (Figure 3D), and demonstrated by cellular fractionation that *SLC25A25-AS1* is enriched in chromatin (Figure 3E). We note that the vast majority of the lncRNAs currently under active investigation satisfy our selection criteria above. This suggests that our experimental design using these three LOF methods is applicable to most studies of lncRNA function.

**Figure 4.**
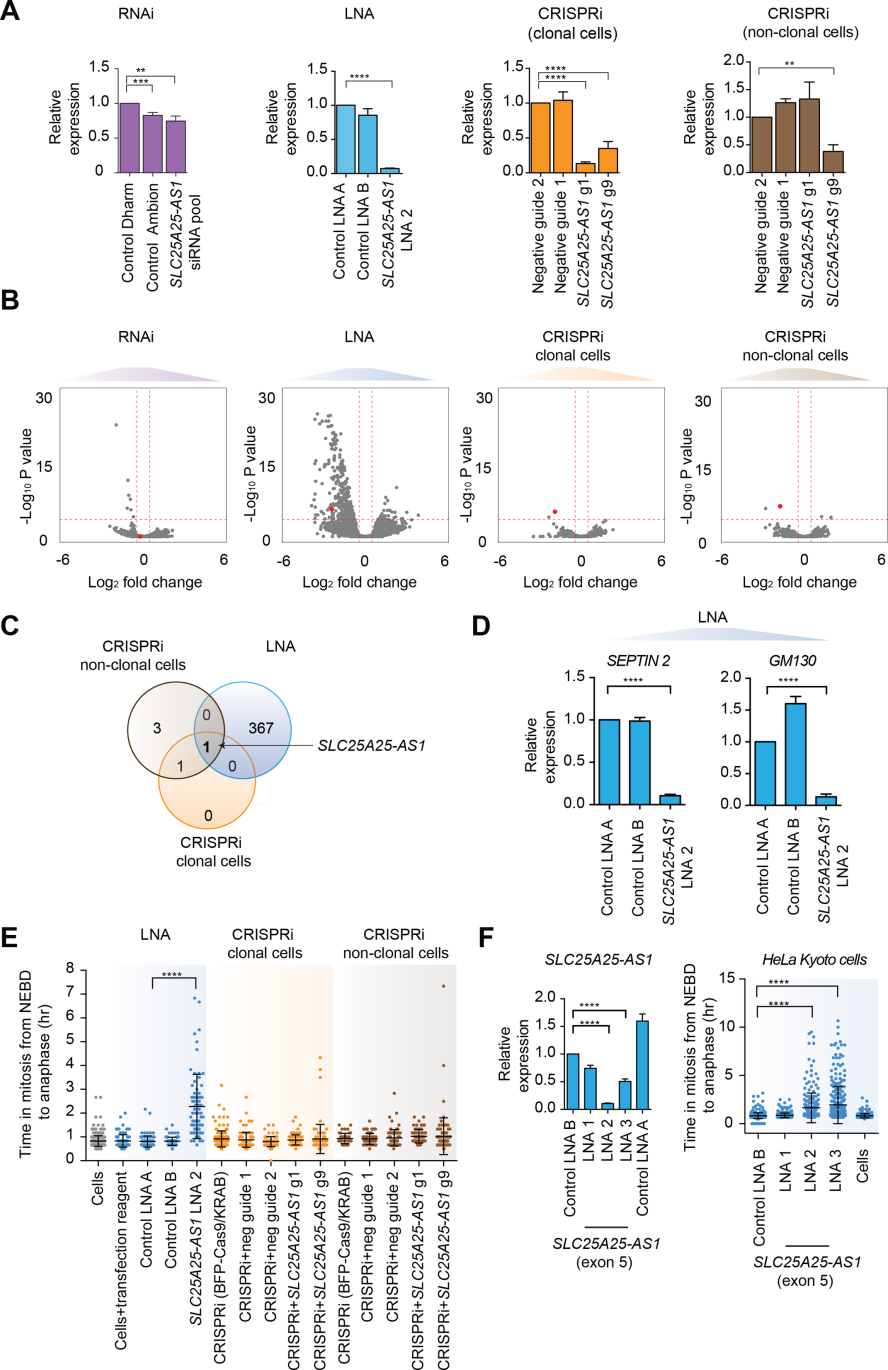
No overlap in DEGs between the different LOF methods upon depletion of *SLC25A25-AS1*. (**A**) Expression levels of *SLC25A25-AS1* after RNAi, LNA and CRISPRi-mediated depletion. qPCR revealed only a 25% reduction in *SLC25A25-AS1* transcription after siRNA-mediated knockdown relative to negative control siRNA from Dharmacon (Control Dharm), and no significant difference relative to negative control siRNA from Ambion (Control Ambion). LNA-mediated knockdown of *SLC25A25-AS1* was performed using LNA oligonucleotide sequence 2 (LNA 2), and showed 90% reduction. CRISPRi-mediated repression of *SLC25A25-AS1* using two guide RNAs targeting the TSS of *SLC25A25-AS1* relative to the negative (non-targeting) guide RNA 2 yielded 70–90% knockdown in clonal cells. Only one guide RNA (guide 9) was efficient in depleting *SLC25A25-AS1* in non-clonal cells. Statistical significance by two-tailed Student's *t*-test: **P< 0.01, ***P< 0.001 and ****P< 0.0001. For all graphs, expression levels of *SLC25A25-AS1* were measured by qPCR using primers spanning exons 1–4, and normalized to the geometric mean of *GAPDH* and *RPS18*. Error bars, s.e.m. (*n* = 4 biological replicates). (**B**) Volcano plots of transcriptional differences induced by RNAi, LNA and CRISPRi-mediated depletion of *SLC25A25-AS1*. After siRNA-mediated depletion of *SLC25A25-AS1*, seven genes were differentially expressed compared to the negative control siRNAs. LNA-mediated depletion with LNA 2 identified 370 DEGs compared to negative control oligonucleotides. CRISPRi-mediated depletion of *SLC25A25-AS1* using guide RNA 1 and 9 revealed only two DEGs compared to negative guide RNA 2. In non-clonal cells, only four DEGs were identified using guide RNA 9 compared to the negative guides. The red horizontal line represents the significance threshold corresponding to an FDR of 5%. Red vertical lines are log_2_-fold change thresholds of ±0.5. The red dot corresponds to the *SLC25A25-AS1* itself. (**C**) Venn diagram showing no overlap between the sets of DEGs identified after using LNA and CRISPRi to deplete *SLC25A25-AS1*. The only gene in common between LNA and CRISPRi-mediated depletion is *SLC25A25-AS1* itself. The total number of genes used for this analysis was 18224. (**D**) qPCR confirmation of the downregulation of two DEGs (*SEPTIN2* and *GM130*) identified in B after LNA-mediated depletion of *SLC25A25-AS1* with LNA 2. Expression levels were normalized to the geometric mean of *GAPDH* and *RPS18*. Error bars, s.e.m. (*n* = 3 biological replicates). Statistical significance by two-tailed Student's *t*-test: ****P< 0.0001. (**E**) Quantification results from time-lapse microscopy of mitotic progression of HeLa cells incubated with Sir-Hoechst after LNA and CRISPRi-mediated (clonal and non-clonal) depletion of *SLC25A25-AS1*. Mitotic duration was measured from nuclear envelope breakdown (NEBD) to anaphase onset. Bars show mean±s.d. (*n* = 2 independent biological replicates). Statistical significance by Mann–Whitney test: ****P< 0.0001. (**F**) *Left panel*: Expression analysis of *SLC25A25-AS1* by qPCR using two additional LNAs targeting *SLC25A25-AS1* (LNA 1 and LNA 3). Expression levels were normalized to the geometric mean of *GAPDH* and *RPS18*. Error bars, s.e.m. (*n* = 3 biological replicates). Statistical significance by two-tailed Student's *t*-test: ****P< 0.0001. *Right panel*: Quantification results from time-lapse microscopy using HeLa Kyoto cells after depletion of *SLC25A25-AS1* using three different LNAs. Bars show mean±s.d. (*n* = 2 biological replicates). Statistical significance by Mann–Whitney test: ****P< 0.0001. The list of DEGs identified after RNAi, LNA and CRISPRi-mediated depletion of *SLC25A25-AS1* are shown in Supplementary Table S2.

We then used the different LOF methods to identify the transcript(s) robustly regulated by *SLC25A25-AS1*, regardless of the method used for depletion. Only modest reduction of *SLC25A25-AS1* levels was observed when the pool of four siRNA sequences was used (Figure 4A). This is in agreement with a previous study showing limited effectiveness of RNAi for depleting nuclear lncRNAs (16). In contrast, we were able to obtain LNA oligonucleotides and CRISPRi guides that achieved over 50% depletion (Figure 4A, B; Supplementary Figure S16). As such, we discarded the RNAi results and attempted to identify a common set of DEGs that were detected with LNA and CRISPRi (clonal and non-clonal cells). The only transcript common to these methods was *SLC25A25-AS1* itself (Figure 4C, Supplementary Table S2). However, the depletion of *SLC25A25-AS1* with LNA resulted in hundreds of DEGs, whereas fewer DEGs were observed with CRISPRi-based methods.

The fact that *SLC25A25-AS1* is the only gene transcriptionally impacted by depletion with LNA and CRISPRi suggests two possible explanations for our results: (i) *SLC25A25-AS1* has no function in transcriptional regulation, the lack of detection with the CRISPRi-based methods is correct, and the DE genes identified with LNA knockdown are off-target effects; or (ii) *SLC25A25-AS1* does regulate the transcription of other genes, the LNA method correctly identifies its downstream targets, and the CRISPRi-based methods are somehow failing to recapitulate the effect. These two explanations are mutually exclusive and cannot be easily distinguished, as the underlying problem stems from the deficiencies of our experimental tools for perturbing the biological system.

We also examined whether the observed transcriptional changes result in a cellular phenotype upon *SLC25A25-AS1* knockdown in HeLa cells. We noticed that two of the top DEGs upon LNA-mediated knockdown of *SLC25A25-AS1*, namely *SEPTIN2* and *GM130* (Figure 4D), had known roles in mitosis (65,66). Depletion of *SEPTIN2* leads to mitotic delay and incomplete cytokinesis whereas inhibition of *GM130* function by antibody injection leads to mitotic delay and multipolar division. Therefore, we assayed if mitotic delay also occurred as a result of *SLC25A25-AS1* depletion. To investigate this, we quantified the time required for HeLa cells to transition through mitosis before and after knockdown of *SLC25A25-AS1*. We observed a significant mitotic delay after LNA-mediated knockdown of *SLC25A25-AS1* (Figure 4E), whereas no such effect was observed with the CRISPRi-based methods. We further confirmed the mitotic delay with an additional LNA oligonucleotide (Figure 4F) in HeLa Kyoto cells, a cell line stably expressing EGFP-α tubulin and mCherry-histone H2B (36). This demonstrates that the differences in the genes disrupted by each method have real consequences on the inferred biological function. Using LNA oligonucleotides, one might conclude that *SLC25A25-AS1* regulates mitosis, whereas the same conclusion cannot be made with CRISPRi.

### Evaluation of on-target activity for each LOF method with protein-coding and non-coding genes

**Figure 5.**
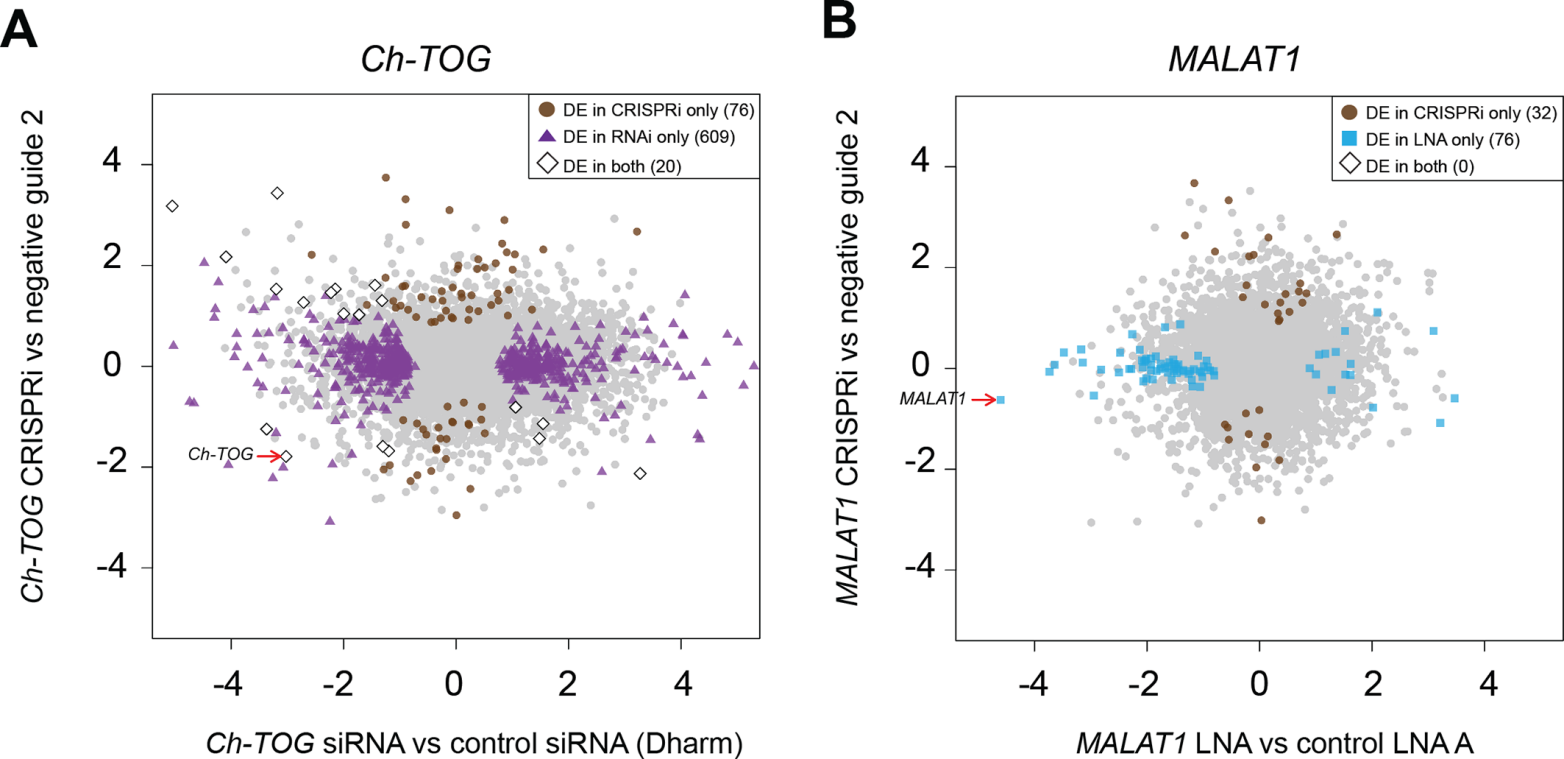
Comparison of DEGs detected upon depletion of well-characterized target genes with different LOF methods. (**A**) Log_2_-fold changes of all genes upon depletion of *Ch-TOG* with CRISPRi (compared to negative guide 2), plotted against the log_2_-fold changes upon depletion with RNAi (compared to negative control siRNA Dharmacon). DEGs in each comparison are highlighted. (**B**) Log_2_-fold changes of all genes upon depletion of *MALAT1* with CRISPRi (compared to negative guide 2) plotted against the log_2_-fold changes upon depletion with LNA (compared to negative control LNA A). DEGs in each comparison are highlighted. In all comparisons, DEGs were defined at a FDR of 5% after testing for a log_2_-fold change significantly greater than 0.5. Lists of DEGs for each comparison are shown in Supplementary Table S3.

We also performed RNA-seq to determine the transcriptional effect of depleting *Ch-TOG* with RNAi and CRISPRi. As previously mentioned, we achieved strong depletion and successfully recovered a well established mitotic phenotype with both LOF methods (Supplementary Figures S1 and S9). As *Ch-TOG* represents a target gene with an essential role in cell division, its depletion should result in a large number of DEGs driven by biology rather than off-target effects. Depletion with RNAi resulted in 693 DEGs compared to the negative control (GE Dharmacon) while depletion of *Ch-TOG* with CRISPRi yielded 87 DEGs compared to a negative guide control (Figure 5A; Supplementary Figure S17A, B; Supplementary Table S3). However, only 20 DEGs were detected with both methods, and of these, only five changed in the same direction - one of which was *Ch-TOG* itself. This is close to the expected number of shared genes between the two sets (2.8 genes) if DEGs were randomly sampled from the pool of all genes, indicating that there is no common transcriptional signature after *Ch-TOG* knockdown. Such a result is unexpected as depletion with both CRISPRi and RNAi yield the expected mitotic phenotype, yet the two methods result in DEG sets that are almost mutually exclusive.

**Figure 6.**
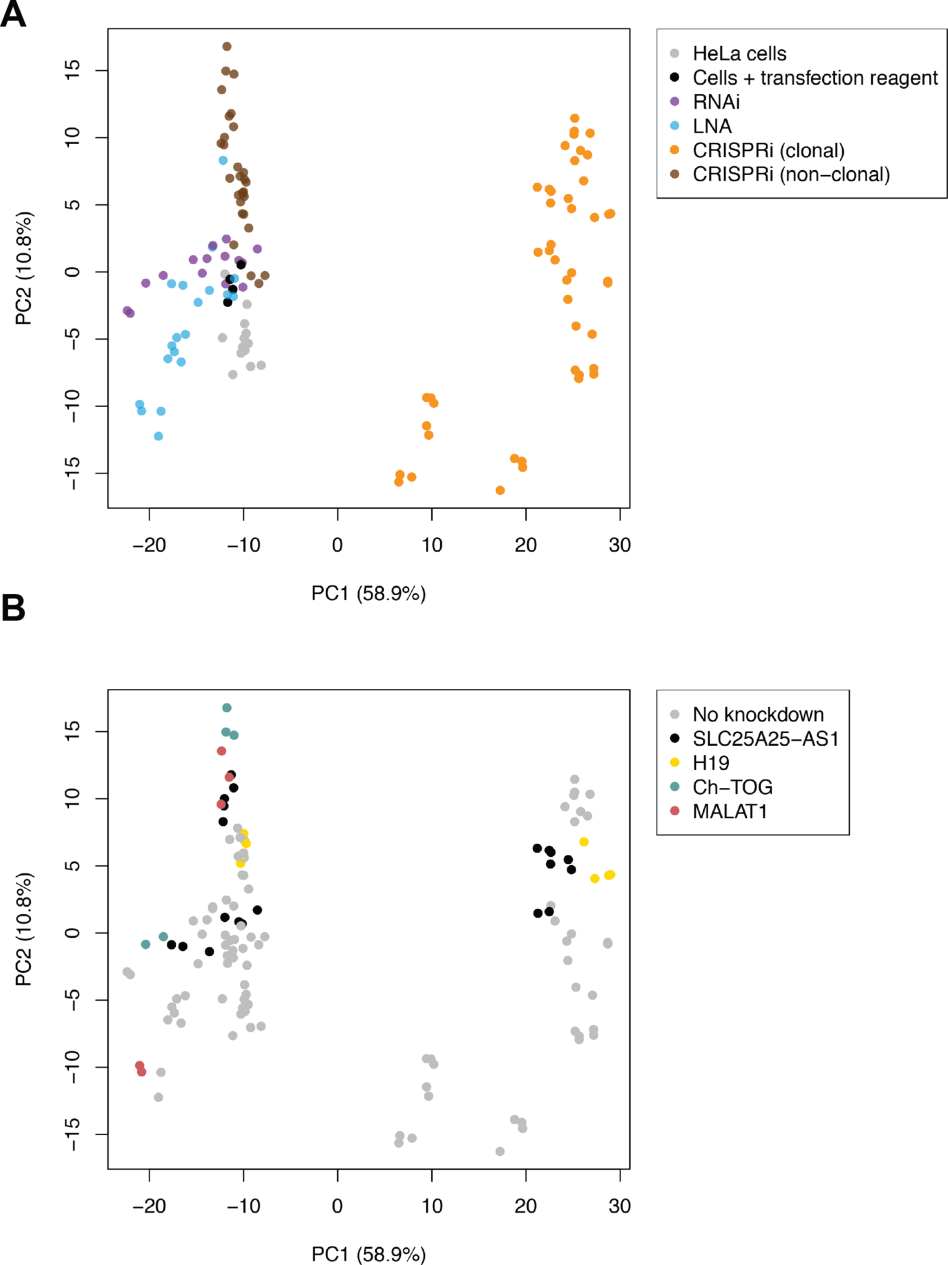
Variation in the gene expression data across samples is driven by the LOF method and not by the identity of the gene being depleted. A principal components analysis (PCA) was performed on the log-expression values of all samples, generated using the top 1000 genes with the lowest *P*-values in a ANOVA-like comparison across all conditions. The top two principal components (PCs) are shown and the numbers in parentheses indicate the proportion of variance explained by each axis. (**A**) PCA plot where each point is coloured by the LOF method used in the corresponding sample. (**B**) PCA plot where each point is coloured by the gene to be depleted.

We observed similar RNA-seq results after depletion of *MALAT1* with LNAs and CRISPRi. One LNA oligonucleotide (positive control, antisense LNA Gapmer LG00000003, Exiqon) was able to effectively deplete *MALAT1* (Supplementary Figure S2B), recapitulating a previously described role of *MALAT1* in mitotic progression (26) (Supplementary Figure S2C and D). By comparison, none of the CRISPRi guides that we tested (five in total) achieved >50% knockdown (Supplementary Figure S18). Unsurprisingly, we detected more DEGs with LNA-mediated depletion compared to the most effective CRISPRi guide (guide 84) (Figure 5B, Supplementary Figure S17C, D; Supplementary Table S3). However, depletion with CRISPRi still yielded a comparable number of DEGs, none of which were present in the set of DEGs detected after depletion with LNA approach. These results are consistent with the discrepancies between the LOF methods that were observed for *SLC25A25-AS1* and *Ch-TOG*, and are most easily explained by strong sequence-dependent off-target effects in either or both LOF methods being compared for each gene. As we have discussed for lncRNA *SLC25A25-AS1*, though, it is not straightforward to determine which LOF method is correct. Indeed, the largest source of variability in expression across our entire dataset was the depletion technology (namely, the effect of single cell cloning in CRISPRi), rather than the target gene that was depleted (Figure 6).

We also used CRISPRi alone to deplete *H19*, a multifunctional and well-characterized lncRNA with activity in the nucleus and in the cytoplasm (67,68). Knockdown efficiency was similar in clonal and non-clonal cells (Supplementary Figure S19A), though there were modest differences in the number of DEGs by RNA-seq. Specifically, we observed 5 and 29 DEGs in clonal and non-clonal populations, respectively, compared to cells treated with negative guides (Supplementary Figure S19B; Supplementary Table S3). This difference hints at the presence of compensatory mechanisms in clonal cells that may be countering dCas9–KRAB activity, possibly due to changes in the transcriptional background (Supplementary Figure S15B). We also examined the expression of genes previously reported to be regulated by *H19* from experiments using RNAi in different human cell lines (69–71) or genetic deletion in mice (72), but we observed no evidence of differential expression for these genes in HeLa cells after *H19* depletion (Supplementary Figure S19C). This highlights another challenge in using LOF methods to infer the function of lncRNAs, as they often operate in a cell type-specific manner (14,26).

## DISCUSSION

Here, we systematically compared three widely used LOF methods and evaluated the transcriptome-wide changes attributable to each individual method. We describe off-target effects associated with each LOF method that need to be considered when investigating gene function, consistent with previous studies (29–31,33–35,54,73). In particular, we identified large off-target effects in the RNAi and LNA methods, which were highly dependent on the siRNA or LNA oligonucleotide sequence. While CRISPRi was less sensitive to the guide sequence, the introduction of dCas9–KRAB provides another source of off-target effects that can significantly change the transcriptional context in the depleted cells. Single cell cloning of dCas9–KRAB-expressing cells results in strong transcriptional changes even in the absence of guide RNAs, indicating that polyclonal populations should be used for CRISPRi experiments.

Our results suggest that CRISPRI in non-clonal populations of dCas9–KRAB-expressing cells provides the cleanest depletion of the target gene, with the fewest off-target effects (sequence-dependent or otherwise). This is consistent with previous studies demonstrating the superiority of CRISPRi compared to RNAi (8,14,73,74). However, CRISPRi has a number of limitations, especially for investigating the function of lncRNAs. Currently, CRISPRi can not differentiate *cis*- and *trans*-acting functions of RNA transcripts (21), *cis*-mediated regulation related to lncRNA transcription (75–77) and/or enhancer-like functions of some lncRNA loci (78–80). In addition, CRISPRi is not ideal for targeting bidirectional promoters (81) or lncRNAs near other transcriptional units (82), as neighboring genes may be unintentionally repressed. The other LOF methods also have their own specific shortcomings—for example, RNAi is known to be less effective for targeting nuclear lncRNAs (16), while LNA oligonucleotides are expensive and multiple sequences need to be tested to obtain at least one with high knockdown efficiency.

Differences between the three LOF methods can lead to significant differences in the molecular or cellular phenotype after depletion of a gene of interest, as observed in our case study with the lncRNA *SLC25A25-AS1*. This is consistent with previous studies that performed depletion of lncRNAs in *in vitro* and *in vivo* models with different technologies. Depletion of *lincRNA-p21* using genetic deletion and RNAi showed no overlap in the sets of differentially expressed genes even in the same cell line (22,23), leading to different conclusions. Indeed, *lincRNA-p21* was proposed to function as a *trans*-acting lncRNA (22), *cis*-acting lncRNA (23) or even as an enhancer-like lncRNA (83). Furthermore, genetic deletion of the lncRNA *megamind* in zebrafish did not reproduce the *megamind* phenotype induced with morpholinos (24,25). Knockdown of *MALAT1* using antisense oligonucleotides or RNAi had different effects on cell growth and cell cycle (27,50). Even more disturbing is the fact that none of these phenotypes were found in *MALAT1* knockout mice (51), suggesting that the function of *MALAT1* is yet to be fully elucidated (85). The function of the well-studied lncRNA *HOTAIR* is similarly controversial (62,86–88). Different targeting strategies to remove the same lncRNA in mice can also lead to some differences in the cellular phenotypes (89,90). Together, these results clearly demonstrate the challenges in characterizing lncRNA function with existing LOF technologies. Such discrepancies are not limited to lncRNAs, but have been observed when depleting protein-coding genes using RNAi and CRISPR-based methods as well as in high-throughput screens (9,61,74,91,92).

The presence of method-specific off-target effects is the most obvious explanation for the discrepancies between LOF methods. For RNAi, these effects are mediated through ‘microRNA-like’ mechanisms that result in hybridisation and silencing of non-targeted transcripts (29,93). For LNAs, oligonucleotides that hybridize to the wrong transcript will inhibit its translation (for protein-coding genes) or trigger its degradation (33). These hybridisation-based mechanisms are supported by the fact that many of our off-target effects for both RNAi and LNA are sequence-dependent. (We note that a few genes are up-regulated in our control comparisons; we hypothesize that this is due to regulatory relationships with down-regulated genes that are directly bound by the siRNA or LNA.) CRISPRi has fewer off-target effects associated with the guide sequence; however, single cell cloning of dCas9–KRAB-expressing cells in the absence of any guide causes a strong transcriptional perturbation. This is probably driven both by the founder effect of cloning as well as the constitutive availability of the KRAB repressive domain in the nucleus. It is also possible that dCas9–KRAB may use endogenous small RNAs as guides. However, more complex biological explanations for the discrepancies between CRISPRi and the other methods cannot be ruled out, e.g., inhibition of transcription with CRISPRi may have *cis* effects that are not present upon depletion at the transcript level with LNAs or RNAi.

To manage the off-target effects of the LOF methods in functional studies, we recommend generating libraries or assaying phenotypes from cells treated with multiple negative control sequences. This allows researchers to quantify the sequence-dependent off-target effects for their experimental system, which are the most problematic as these effects do not cancel out when comparing between negative control sequences and sequences against the targeted gene. We also recommend the collection of data for negative controls obtained at each step of the method, e.g., untreated cells, cells treated with transfection reagent alone (for RNAi/LNA) or cells expressing dCas9–KRAB without the addition of guide sequence (for CRISPRi). This allows accurate quantification of the extent of off-target effects introduced upon sequential manipulations in the LOF protocol, which can alter the transcriptional or cellular background in which the depletion occurs (potentially resulting in different phenotypes). To our knowledge, this is not common practice in the field, with the vast majority of studies only using a single negative control sequence to determine the molecular or cellular phenotype upon depletion. In transcriptomic studies, genes affected by off-target activity in the negative controls can be excluded from the DE analysis in samples where the gene of interest has been depleted, thus mitigating the impact of off-target effects on the biological conclusions. Results should also be disregarded if the number or log-fold changes of the off-target genes are comparable to or greater than the number or log-fold changes of DEGs detected upon knockdown of the target gene. In such cases, there is no meaningful way to distinguish between off-target effects and genuine downstream targets of the gene of interest.

We also recommend performing differential expression analyses with a minimum log-fold change threshold, to avoid detecting genes with small changes in expression due to off-target effects. The use of a threshold mitigates the effect of non-specific activity on the differential expression results for each LOF method, by focusing on larger and arguably more biologically relevant effects of depletion. Obviously, a more stringent testing procedure will reduce power to detect genuine downstream targets with small log-fold changes. However, false negatives are generally of less concern than false positives in DE analyses where hundreds to thousands of genes are routinely detected with significant changes in expression. Indeed, genes with small log-fold changes are challenging to validate and less appealing for functional studies. This reduces the motivation for using a more relaxed testing procedure, especially if more genes are incorrectly detected due to off-target effects. The use of a log-fold change threshold is fairly widespread in DE analyses of RNA-seq data; nonetheless, in contexts where the effects are known to be weak, one might decide to not use any threshold to favour increasing the detection power over reducing off-target effects.

Another strategy for reducing off-target effects is to use multiple targeting sequences (e.g., multiple siRNAs, LNAs or CRISPRi guides) against the gene of interest. Each sequence should target a different region of the gene, i.e., different exons for siRNAs, exons or introns for LNAs, and different intervals in the promoter region for CRISPRi. Any common effects across targeting sequences are likely to represent genuine biology of depletion of the gene of interest, while sequence-specific effects can be attributed to off-target activity. For example, the DEG sets that are generated after depletion with each targeting sequence can be intersected to identify a reliable set of genuine downstream targets (albeit with reduced detection power, due to the conservativeness of the intersection operation). A recent study has recommended using multiple sequences to obtain a reproducible molecular phenotype (73). We agree but note that it is not always practical to obtain multiple targeting sequences with high depletion efficiency for a gene of interest. For example, our experience with *MALAT1* shows that only one out of five CRISPRi guides achieved over 50% depletion efficiency, while for *SLC25A25-AS1*, only one out of three LNA oligonucleotides achieved over 90% efficiency.

Other experimental factors can also be tuned to control the extent of off-target activity in each LOF method. One potential approach to minimize off-target effects is to titrate the dose of reagents (from 1–30nM for LNAs and 1–50nm for siRNAs) and use the lowest concentration that yields satisfactory depletion of the target gene (16). This may reduce off-target effects by decreasing the opportunity for incorrect hybridization to sequences other than the gene of interest. Indeed, the use of the minimum dose has previously been encouraged (94) and is a prudent strategy for improving the specificity of LOF methods in routine experiments. It may also be informative to assay the molecular and cellular phenotypes at earlier time points (e.g., 6–12 hours), which might reduce the off-target effects by minimizing their propagation throughout regulatory networks. However, this is dependent on the experimental context, as later time points may still be necessary to fully characterize the biological effects of depletion. Indeed, a recent study that performed a CRISPRi screen on lncRNAs observed cell growth defects 10–20 days after their depletion (14). In such cases, even indirect off-target effects are problematic and need to be considered when evaluating depletion specificity.

Another important aspect of functional studies is the validation of molecular and cellular phenotypes with rescue experiments. This is usually done by overexpression of the gene of interest to counter the effect of depletion, e.g., for RNAi, transfection or transduction of exogenous transcripts of the gene that have been modified to be resistant to RNAi-mediated depletion. This ensures that the proposed phenotype can be correctly attributed to the targeted gene, regardless of the off-target effects of the depletion method. We suggest that rescue experiments should be routinely performed for validating gene function after its depletion. However, some care is required during the design of these rescue experiments to ensure that gene expression is restored to physiological levels. Particular care needs to be taken when overexpressing lncRNAs so that their intracellular localization is preserved, as demonstrated for *linc-PINT* (95).

We note that we have only considered a small subset of possible LOF methods that are used by the research community. For RNAi, alternative methods include short hairpin RNAs (96), siPOOL (97) or C911 mismatch siRNAs (98). In the case of LNAs, oligonucleotides can be designed to target intronic regions of gene of interest (99). A variety of other CRISPR-based strategies are available, such as whole locus or promoter deletion, insertion of transcriptional termination sites into the gene body as well as the CRISPR-Cas13 system (100). It remains to be seen whether these methods have similar off-target effects to the protocols we have studied here.

Modern LOF technologies allow researchers to deplete any transcript of interest in a variety of biological systems, and provide an essential experimental toolkit for studying the biological function of transcripts and dissecting networks of transcriptional regulation. Here, we have empirically formulated recommendations to minimize technical artefacts and avoid—or at least prudently manage—the off-target effects of these commonly used LOF methods.

## DATA AVAILABILITY

Sequencing data are available in the ArrayExpress database (http://www.ebi.ac.uk/arrayexpress) under the accession number E-MTAB-5308.

## Supplementary Material

Supplementary Data
