# Supplementary material for: Specificity of RNAi, LNA and CRISPRi as loss-of-function methods in transcriptional analysis
Source: Nucleic Acids Res. 2018 Jun 1;46(12):5950–66. doi: 10.1093/nar/gky437 (PMC6093183; doi:10.1093/nar/gky437)
Supplement: Supplementary Data [file gky437_supplemental_files.zip › StojicLun_Supplementary_20180205.pdf]

Supplementary Data

Specificity of RNAi, LNA and CRISPRi as loss-of-function methods in transcriptional analysis

Lovorka Stojic[1*,†,] Aaron TL Lun[1*,†], Jasmin Mangei[1], Patrice Mascalchi [1], Valentina Quarantotti[1], Alexis R Barr[2], Chris Bakal[2], John C Marioni[1,3,4], Fanni Gergely[1] and Duncan T Odom[1]

[1] Cancer Research UK Cambridge Institute, University of Cambridge, Li Ka Shing Centre, Robinson Way, Cambridge CB2 0RE, UK.
[2] Institute of Cancer Research, 237 Fulham Road London SW3 6JB, UK.
[3] European Bioinformatics Institute, European Molecular Biology Laboratory (EMBL-EBI), Wellcome Genome Campus, Hinxton, Cambridgeshire, CB10 1SD, UK.
[4] Wellcome Trust Sanger Institute, Wellcome Genome Campus, Hinxton, Cambridgeshire, CB10 1SA, UK

* Equal contributions

† To whom correspondence should be addressed

Tel: +44 (0) 1223 769 655; Fax: +44 (0) 1223 769 881
Email: Lovorka.stojic@cruk.cam.ac.uk; aaron.lun@cruk.cam.ac.uk

Present Address: *Patrice Mascalchi*: Bordeaux Imaging Center, UMS 3420 CNRS, US4 INSERM, University of Bordeaux, 33000 Bordeaux, France. *Jasmin Mangei:* German Cancer Research Center (DKFZ), Division: Molecular Genetics, Im Neuenheimer Feld 580, D-69120 Heidelberg

Issue Section: RNA, Genomics, Computational biology

The PDF file includes:
Supplementary methods
Supplementary figures 1-20
Supplementary references

Supplementary methods

Data processing

Reads were aligned to the hg38 build of the human genome using subread v1.5.3[1] in paired-end RNA-seq mode with unique mapping. The sequence of the pHR-SFFV-dCas9-BFP-KRAB vector was also included to ensure proper mapping of reads in libraries derived from dCas9-KRAB-positive cells. The number of read pairs mapped to the exonic regions of each gene was then counted for each library, using the featureCounts function in Rsubread v1.28.0[2] with Ensembl GRCh38 version 90. Only alignments with mapping quality scores above 10 and with the first read pair on the reverse strand were considered during counting. The number of reads mapped to the ~4kbp region coding for the dCas9 domain was also counted in each library for diagnostic purposes. For any sample with multiple technical replicates (due to sequencing across multiple lanes), counts from all technical replicates were summed for each gene prior to further analysis.

Differential gene expression (DE) analysis

The DE analysis was performed in R using methods from the limma package v3.34.1[3]. First, lowly expressed genes with average counts per million (CPMs) below 3 were filtered out, as was dCas9-KRAB itself. Normalization was performed using the trimmed mean of M-values method[4] to remove composition biases. Log-transformed expression values with combined precision/array weights were computed with the voomWithQualityWeights function[5]. The experimental design was parametrized using an additive model with a group factor, where each group was comprised of all samples from one batch/treatment combination; and an experiment factor, representing samples generated on the same day.

Robust empirical Bayes shrinkage[6] was performed using the eBayes function. Testing for DE genes was performed between pairs of groups using the treat function[7]. Here, the null hypothesis was that the absolute $\log_2$-fold change between groups was less than or equal to 0.5. We also repeated the analysis using a moderated t-test, with the null hypothesis being that the $\log_2$-fold change between groups was equal to zero. Almost all pairwise contrasts involved groups from the same batch to avoid spurious differences due to batch effects. If the samples of the groups of interest belonged to different batches, we instead tested for differences in the log-fold change for each group of interest over another group that was present in both batches (i.e. a "differential differences" comparison, where the batch effects are cancelled out by contrasting to a common group within each batch).

Note that some groups are repeated across several batches, such that some (but not all) contrasts can be performed within multiple batches. In such cases, only the results of the contrast of groups in one of the batches were used in downstream analyses. This simplifies the interpretation of the number of DE genes, as this may not be comparable between contrasts if results from multiple batches are merged for some contrasts and not others.

DE analyses were performed separately for the two rounds of experiments that were performed in the

manuscript. The first round of experiments contained all of the samples used in Figures 1-4, i.e., the many negative controls for each LOF method and the depletion experiments for *SLC25A25-AS1* and *H19*. The second round of experiments contained all of the samples used in Figure 5, i.e., the depletion experiments for *Ch-TOG* and *MALAT1.*

Construction of the flow charts

Adjusting the p-values for multiple testing was performed using the Benjamini-Hochberg (BH) correction to control the false discovery rate (FDR) at 5%. For all flowcharts, the p-values from all pairwise contrasts in all flowcharts were pooled. The BH correction was then applied to the pooled values. This ensures that a consistent significance threshold is applied to all contrasts, allowing the numbers of DE genes to be directly compared between contrasts. (Note that this principle also motivates the fitting of a single model with a shared variance estimate for all groups, to mitigate any differences in detection power between comparisons.)

Construction of the volcano plots

In the volcano plots for the comparisons between the negative controls, the p-value threshold was defined as that corresponding to the FDR of 5% used in the flow charts.

To generate a volcano plot for depletion of *SLC25A25-AS1* with each LOF method, the DE results from the two lncRNA depletion contrasts (i.e., siRNA versus negative siRNA control Ambion or negative siRNA control GE Dharmacon for RNAi; LNA 2 versus negative control LNA A or B for LNAs; *SLC25A25-AS1* guide 1 or 9 versus negative guide 2 for CRISPRi clones; and *SLC25A25-AS1* guide 9 versus negative guide 1 or 2 for CRISPRi non-clonal cells) were first combined. This was performed using an intersection-union test[8], where the combined p-value for each gene was defined as the larger p-value from the two contrasts. This means that a gene is only considered to be DE if it is significant in both contrasts. The combined $\log_2$-fold change for each gene was defined as the average of the $\log_2$-fold changes from the two contrasts. To set the p-value threshold in each plot, combined p-values were pooled from all methods and the BH method was applied to control the FDR across the pool at 5%. The threshold was then defined as the largest p-value in the set of significant genes. This approach is based on the same reasoning as described for the flowcharts and yields a constant threshold for intuitive comparison between plots for different LOF methods. The same merging strategy and threshold were also applied to the *H19* results to obtain volcano plots for *H19* depletion.

To generate a volcano plot for depletion of *Ch-TOG* with CRISPRi or RNAi, all p-values from the relevant comparisons were pooled and the BH method was applied to control the FDR across the pool at 5%. The p-value threshold in each volcano plot was defined as the largest p-value in the set of significant genes. The same strategy was used to create volcano plots after depletion of *MALAT1* with CRISPRi or LNA oligonucleotides.

Construction of the Venn diagrams

To generate the Venn diagram after *SLC25A25-AS1* depletion with the different LOF methods, DEGs from each method were defined as those with combined p-values below the threshold defined in the volcano plots. The number of genes in each intersection was calculated using the vennDiagram function from the limma package. To generate the Venn diagram of DEGs between CRISPRi clones and parental cells, p-values were pooled from all contrasts and the BH method was applied to control the FDR at 5%. (Again, this ensures that numbers are directly comparable between contrasts.) The resulting sets of DEGs for all clone-parent comparisons were used to calculate the numbers in the intersections of the Venn diagram.

Construction of the MA plots

To construct an MA plot for a given pairwise comparison, DEGs were defined using an appropriate p-value threshold (from the flow charts for comparisons between negative controls, or using the threshold defined in the volcano plots for comparisons involving depletion of target genes). The log-fold changes (M) and average abundances (A) for all non-DEGs were visualized using the smoothScatter function in R, where the intensity of colour is proportional to the density of points. DEGs were visualized as separate points, coloured by the direction of change. For all non-DEGs and DEGs changing in each direction, the distribution of log-fold changes and average abundances were also shown using boxplots.

Construction of the PCA plots

To correct for batch effects, we equalized the average log-expression of shared experimental groups across batches. This mainly involved scaling the expression of all batches so that the average expression in the group of untreated cells for each batch was the same as that in the first batch. The same approach was used for batches that did not contain untreated cells, instead using the average expression for the CRISPRi clone 2 group (without any guide treatment) or that of the method-specific negative controls.

To select features of interest, we performed a DE analysis on all samples using the additive model described above. We performed a moderated F-test (i.e., an ANOVA-like comparison) to test for any differences between any groups, and selected the top 1000 genes with the smallest p-values. The log-expression profiles of these genes were used to perform a principal components analysis (PCA) using the prcomp function in R. The top two principal components were used for visualization of the differences between samples.

KEGG gene set analyses

To perform KEGG enrichment analyses for each contrast, Ensembl identifiers were first converted to Entrez identifiers using the org.Hs.eg.db package. The set of DEGs for each contrast was the same as those in the flowcharts, except for genes that were removed because they did not have Entrez identifiers. The kegga function was then applied to test for significant overrepresentation of KEGG terms in the set of DEGs. This analysis was also applied to the common set of DEGs present in all

comparisons between clones and parental cells.

Integration with dCas9 ChIP-seq and RNA-seq data

We obtained BED files containing dCas9 binding sites in 293T cells from the NCBI Gene Expression Omnibus under the accession numbers GSM1496583 and GSM1496584 [9]. For our common set of DEGs present in all clone-parental, we extracted their genomic coordinates using the hg19 Ensembl annotation (we used hg19 here, rather than hg38 as performed in the analyses above, as the coordinates reported by *O'Geen et al.,* [9] were generated by analyses based on the hg19 genome build). Any overlaps between the dCas9 binding sites and the gene bodies or their 10 kbp flanking regions were identified using the findOverlaps function in the GenomicRanges package. We also obtained dCas9-KRAB ChIP-seq data generated by *Thakore et al.,* [10] in K562 cells from NCBI GEO (GSM1819865, GSM1819866, GSM1819867). Each library was aligned to the hg38 genome using subread in genomic alignment mode with unique mapping. Peaks were called using MACS2 aligner[11] and high-confidence binding sites were selected as those with a peak-calling score above 10. These sites were converted into hg19 coordinates using the liftOver function from the rtracklayer package[12] and overlapped with the gene bodies as previously described. Finally, we obtained the list of genes that were significantly DE upon dCas9-KRAB transduction from the supplementary materials of *Thakore et al.,* [10], and identified the intersection between their genes and our common set.

Code availability

All code used in this analysis is available at https://github.com/MarioniLab/LOFMethods2016.

Supplementary Table 5. List of RNAi sequences.

siRNA	Sequence (Antisense)	Product number
siGENOME Non-Targeting siRNA Pool #2 (GE Dharmacon)	/	001206-14-20
Negative control siRNA #1 (Thermo Fischer Scientific, Silencer select, Ambion)	/	4390084
SLC25A25-AS1 Lincode Smart pool siRNA target 1 (GE Dharmacon)	GGGAUUACCUGGAGCGUGU	R-188737-00-0005, *N-188737-01*
SLC25A25-AS1 Lincode Smart pool siRNA target 2 (GE Dharmacon)	GGAAGUAGCUUAAACAAUG	R-188737-00-0005, *N-188737-02*
SLC25A25-AS1 Lincode Smart pool siRNA target 3 (GE Dharmacon)	GCACCCAGGUAUAGUUAGU	R-188737-00-0005, *N-188737-03*
SLC25A25-AS1 Lincode Smart pool siRNA target 4 (GE Dharmacon)	GCUUCACAGACUCGGGAAU	R-188737-00-0005, *N-188737-04*
CKAP5 (Ch-TOG) On-targetplus SMARTpool (GE Dharmacon)		L-006847-00
MALAT1 Lincode Smart pool (GE Dharmacon)		R-187978-00-0005

Supplementary Table 6. List of LNA Gapmer sequences (Exiqon).

**LNA**	**sequence (5'to3')**	**Product number**
Negative control A	AACACGTCTATACGC	300611-00
Negative control B	GCTCCCTTCAATCCA<br>A	300615-00
SLC25A25-AS1<br>*LNA Gapmer no2*<br>*(Exon 5)*	TTTGGTGAGAGAAAT<br>C	300600<br>Design ID: 380340-2
SLC25A25-AS1<br>*LNA Gapmer no1*<br>*(Exon 5)*	TACTCAGCAACGAAT<br>G	300600<br>Design ID: 572606-1
SLC25A25-AS1<br>*LNA Gapmer no3*<br>*(Exon 5)*	CAGCGGGACACGTGA<br>G	300600<br>Design ID: 572606-3
MALAT1<br>LNA Gapmer		3000601-00<br>positive control

Supplementary Table 7. List of CRISPRi guide RNA sequences.

Targeted lncRNA	Guide-ID	Guide sequence	Reference
SLC25A25-AS1	sgRNA 1	GATGGAGAATGTAAGGGTAC	
	sgRNA 9	TGCAGAGAACGGAGGCATGC	
H19	sgRNA 2	GCTAGGACCGAGGAGCAGGGTG	*Gilbert et al., 2014*
Negative Control	sgRNA 1	GCGCCAAACGTGCCCTGACGG	*Gilbert et al., 2014*
Negative Control	sgRNA 2	GTGCGATGGGGGGGTGGGTAGC	*Gilbert et al., 2014*
Ch-TOG/CKAP5	sgRNA 94	TAAGCCGTTTGAAACCGCTT	
MALAT1	sgRNA 86	AAGGACTGGGGCCCCGCAAC	
MALAT1	sgRNA 93	AAAATGGCGCTGCGCTTAAG	
MALAT1	sgRNA 84	GCAGCCCGAGACTTCTGTAA	
MALAT1	sgRNA 1		*Gilbert et al., 2014*
MALAT1	sgRNA 2		*Gilbert et al., 2014*

Supplementary Table 8. List of primer sequences for qPCR.

**Expression primers**	**Forward primer (5'to3')**	**Reverse primer (5'to3')**
GAPDH	CAACAGCCTCAAGATCATCAG	ATGGACTGTGGTCATGAGTC
RPS18	ATCCCTGAAAAGTTCCAGCA	CCCTCTTGGTGAGGTCAATG
β-ACTIN	GTTACACCCTTTCTTGACAAA	GTCACCTTCACCGTTCCAGTT
H19	CTGGCTTGGCAGACAGTACA	TCCCTCCTGAGAGCTCATTC
MALAT1	GACGGAGGTTGAGATGAAGC	ATTCGGGGCTCTGTAGTCCT
SLC25A25-AS1 (exon 1-4)	CACCTAGGCCCAGCTTCTC	TTCAGACACGCTCCAGGTAA
SCIN QT00095179 (QIAGEN)	/	/
CDH2 QT00063196 (QIAGEN)	/	/
EXOC4 QT00040208 (QIAGEN)	/	/
UBA5 QT00039067 (QIAGEN)	/	/
DICER1 QT00015176 (QIAGEN)	/	/
ITGA2 QT00086695 (QIAGEN)	/	/
SEPT2 QT00056105 *(QIAGEN)*	/	/
GM130 (GOLGA2) QT00028392 *(QIAGEN)*	/	/
CKAP5 (Ch-TOG) QT00047054 *(QIAGEN)*	/	/

Supplementary Figures

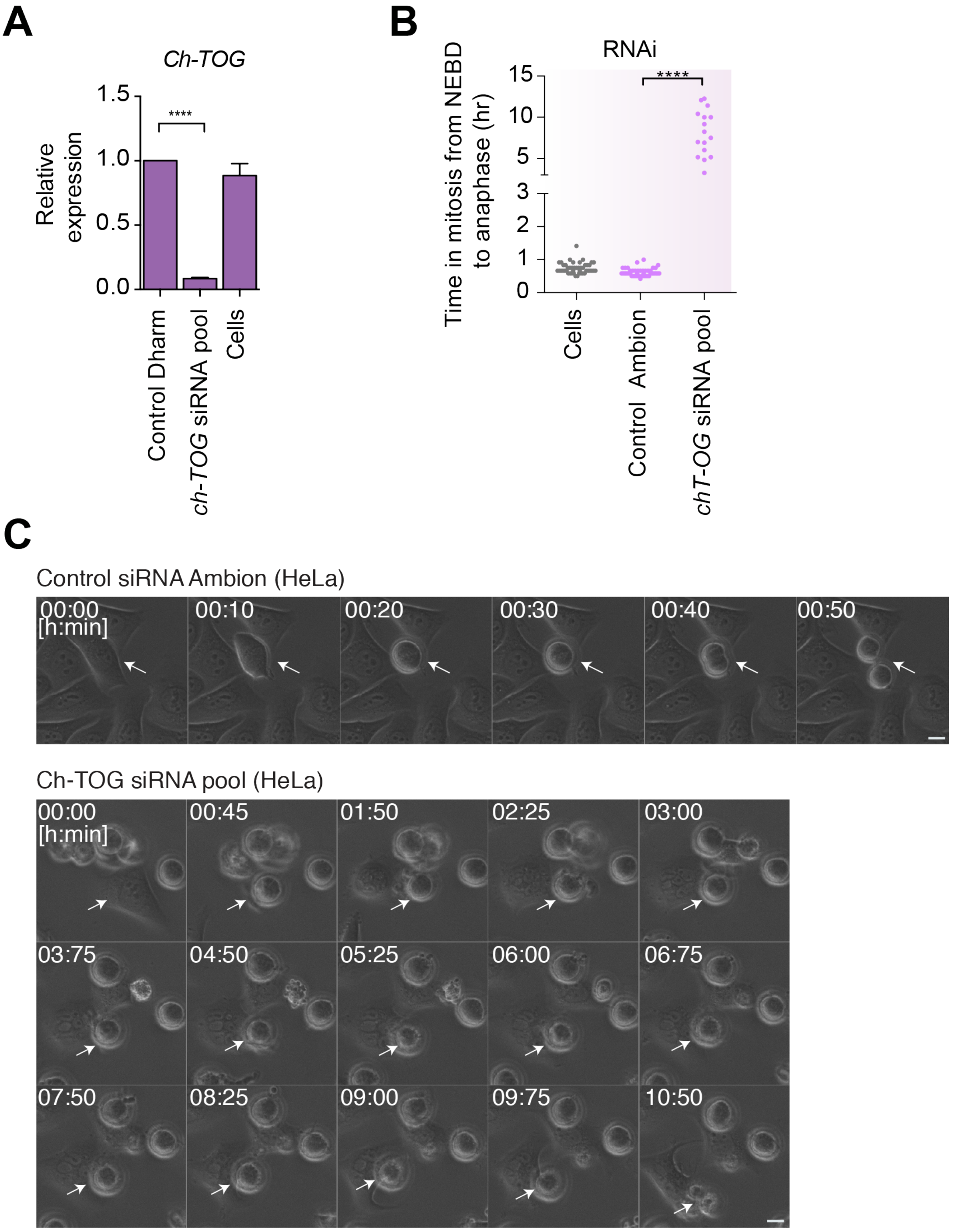

Supplementary Figure 1. Depletion of *Ch-TOG* using RNAi leads to mitotic delay.

- **A.** Expression of *Ch-TOG* was quantified by qPCR after RNAi-mediated depletion using pool of four *Ch-TOG* siRNA sequences. Expression levels were normalized to the geometric mean of *GAPDH* and *RPS18.* Error bars, s.e.m. (*n*=3 biological replicates). Statistical significance by two-tailed Student's *t*-test: **** P<0.0001.
- **B.** Quantification results from time-lapse microscopy of mitotic progression of HeLa cells after RNAi-mediated depletion of *Ch-TOG.* Bars show mean (*n*=one biological replicate).

Statistical significance by Mann-Whitney test: ****$P<0.0001$. Mitotic duration was measured from nuclear envelope breakdown (NEBD) to anaphase onset.

C. Representative still images from the time-lapse microscopy. White arrows depict cell going through mitosis. Scale bar indicates 20µm.

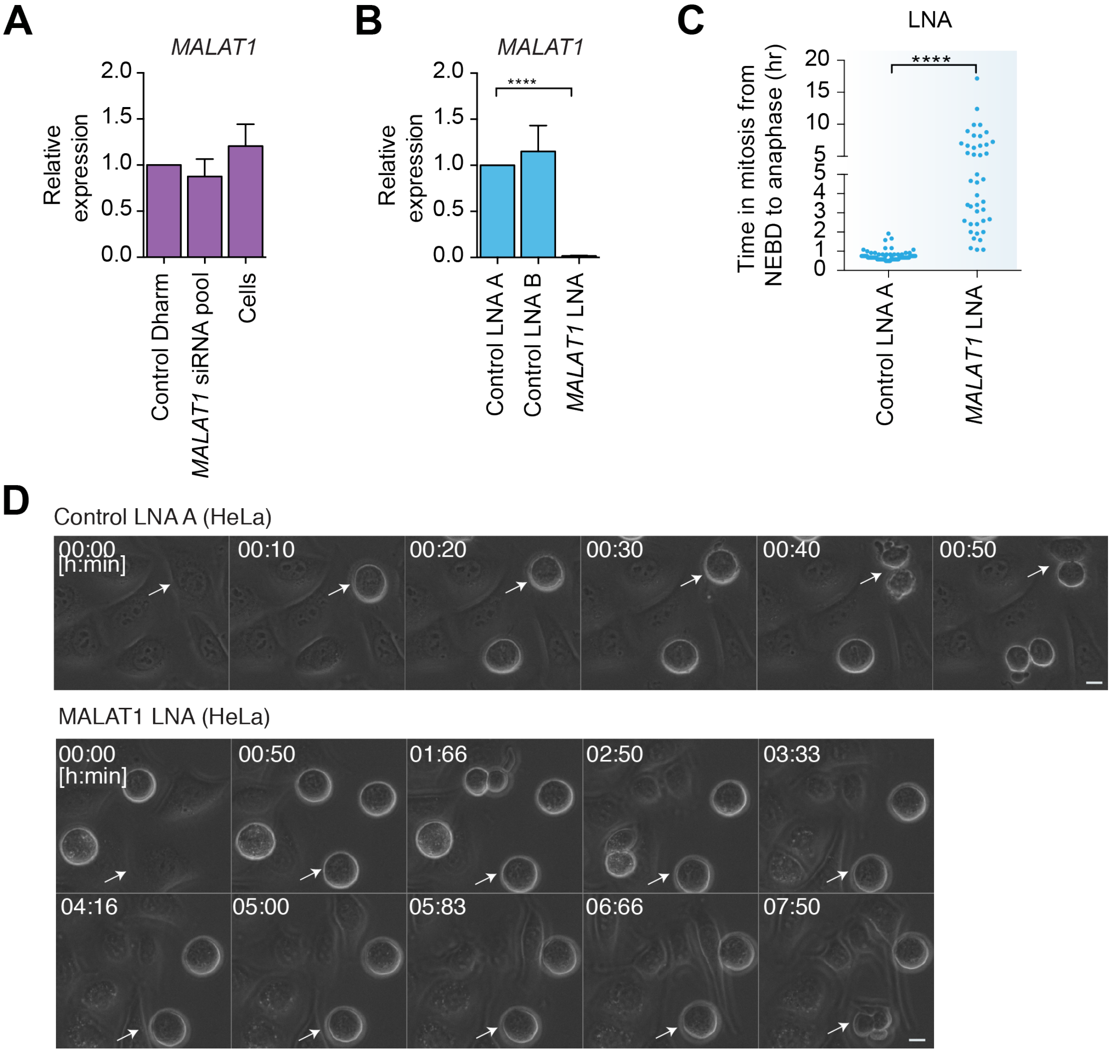

Supplementary Figure 2. Depletion of lncRNA *MALAT1* using RNAi, LNA and CRISPRi.

A-B. Expression of *MALAT1* was quantified by qPCR after RNAi (A) and LNA-mediated depletion of *MALAT1* (B). The pool of siRNA sequences did not deplete *MALAT1*. In both cases, expression levels were normalized to the geometric mean of *GAPDH* and *RPS18.* Error bars, s.e.m. (*n*=3 biological replicates). Statistical significance by two-tailed Student's *t*-test: **** P<0.0001.

C. Quantification results from time-lapse microscopy of mitotic progression of HeLa cells after LNA-mediated depletion of *MALAT1.* Bars show mean (*n*=one biological replicate). Statistical significance by Mann-Whitney test: ****P<0.0001. Mitotic duration was measured from nuclear envelope breakdown (NEBD) to anaphase onset.

D. Representative still images from the time-lapse microscopy. White arrows depict cell going through mitosis. Scale bar indicates 20μm.

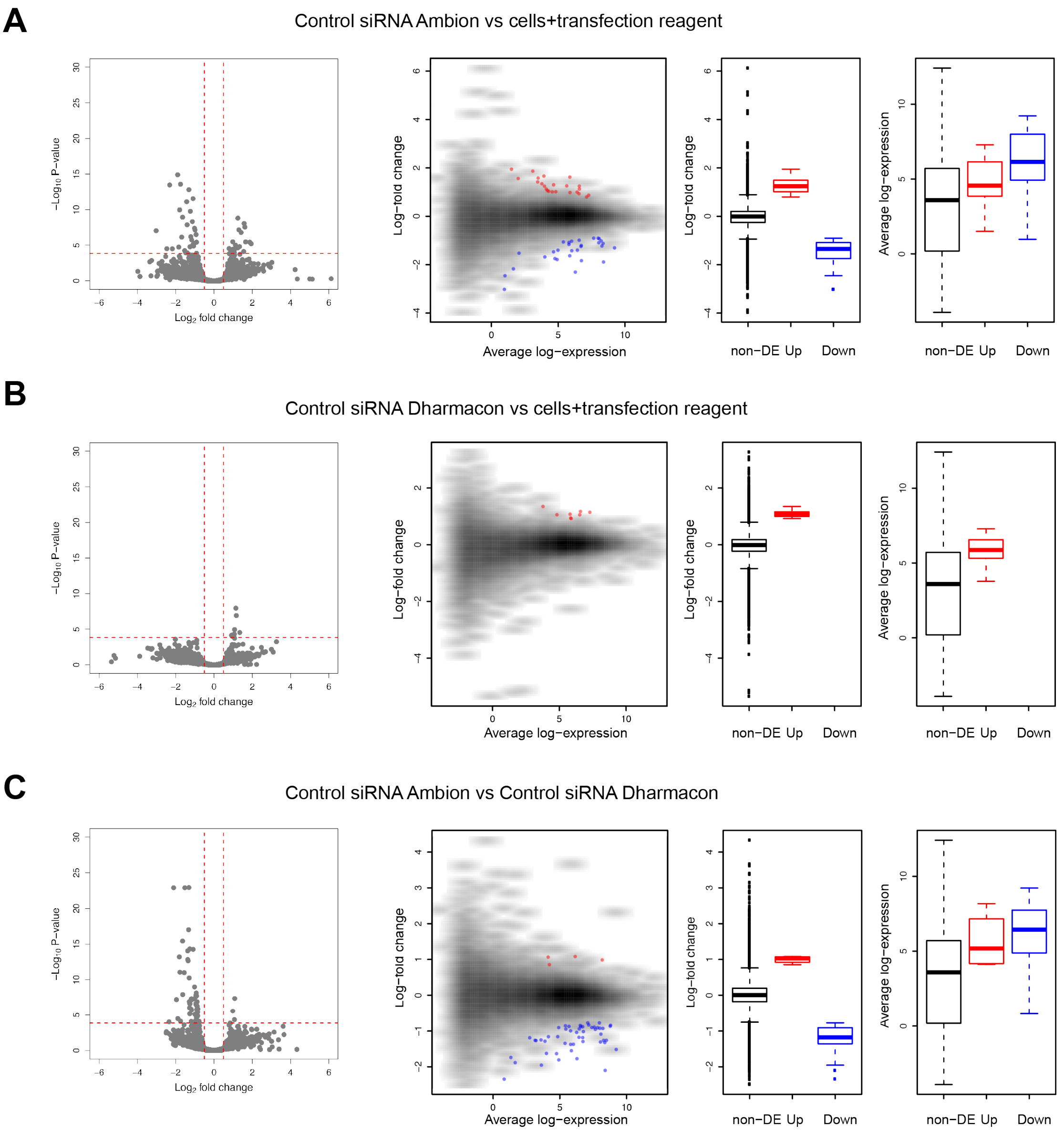

Supplementary Figure 3. Statistics of DEGs between RNAi negative controls.

A volcano plot (left panel) and MA plot (centre panel) is shown for each pairwise comparison in Figure 1A. DEGs were detected at a FDR of 5% (corresponding to the dashed horizontal line in each volcano plot) after testing against a log-fold change threshold of 0.5 (dashed vertical lines). For the MA plot, the intensity of colour is proportional to the density of non-significant genes, while DEGs are highlighted as separate points. The distribution of log-fold changes and average abundances for all DEGs changing in each direction are shown in the boxplots (right panels), along with the distributions for non-significant genes.

A. Comparison between cells treated with the negative control siRNA (Ambion) and cells treated with the transfection reagent.

B. Comparison between cells treated with the negative control siRNA (GE Dharmacon) and cells treated with the transfection reagent.

C. Comparison between cells treated with the negative control siRNA (Ambion) and cells treated with the negative control siRNA (GE Dharmacon).

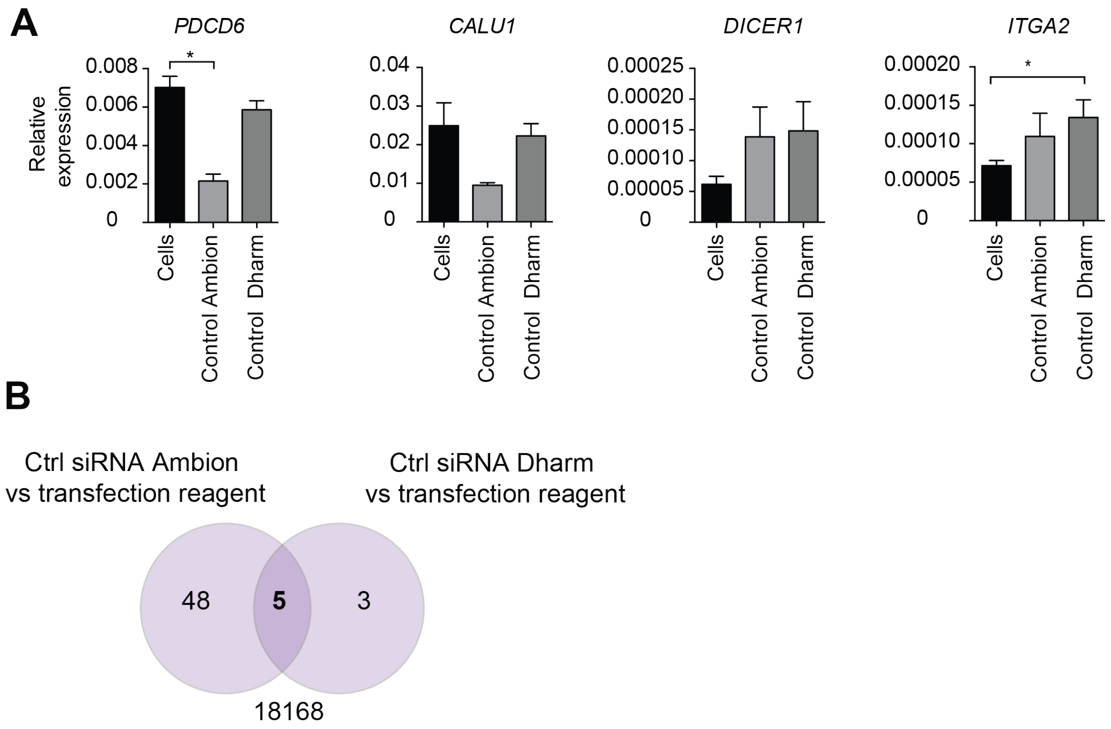

Supplementary Figure 4. Validation of off-target changes in gene expression in the RNAi approach using negative control siRNAs from Ambion or GE Dharmacon (Dharm).

A. Expression of genes (*PDCD6, CALU1, DICER1, ITGA2*) affected by transfection of negative control siRNAs compared to HeLa untreated cells. Expression was measured by qPCR and normalized to the geometric mean of *GAPDH* and *RPS18.* Error bars, s.e.m. (n=3 biological replicates). Statistical significance by two-tailed Student's t-test: * P<0.1.

B. Overlap between DEGs detected in the comparison of cells treated with each negative control siRNA versus cells treated with transfection reagent. Each set of DEGs is the same as that defined in Figure 1 for the corresponding pairwise comparisons. The total number of genes in the analysis is shown outside of the Venn diagram.

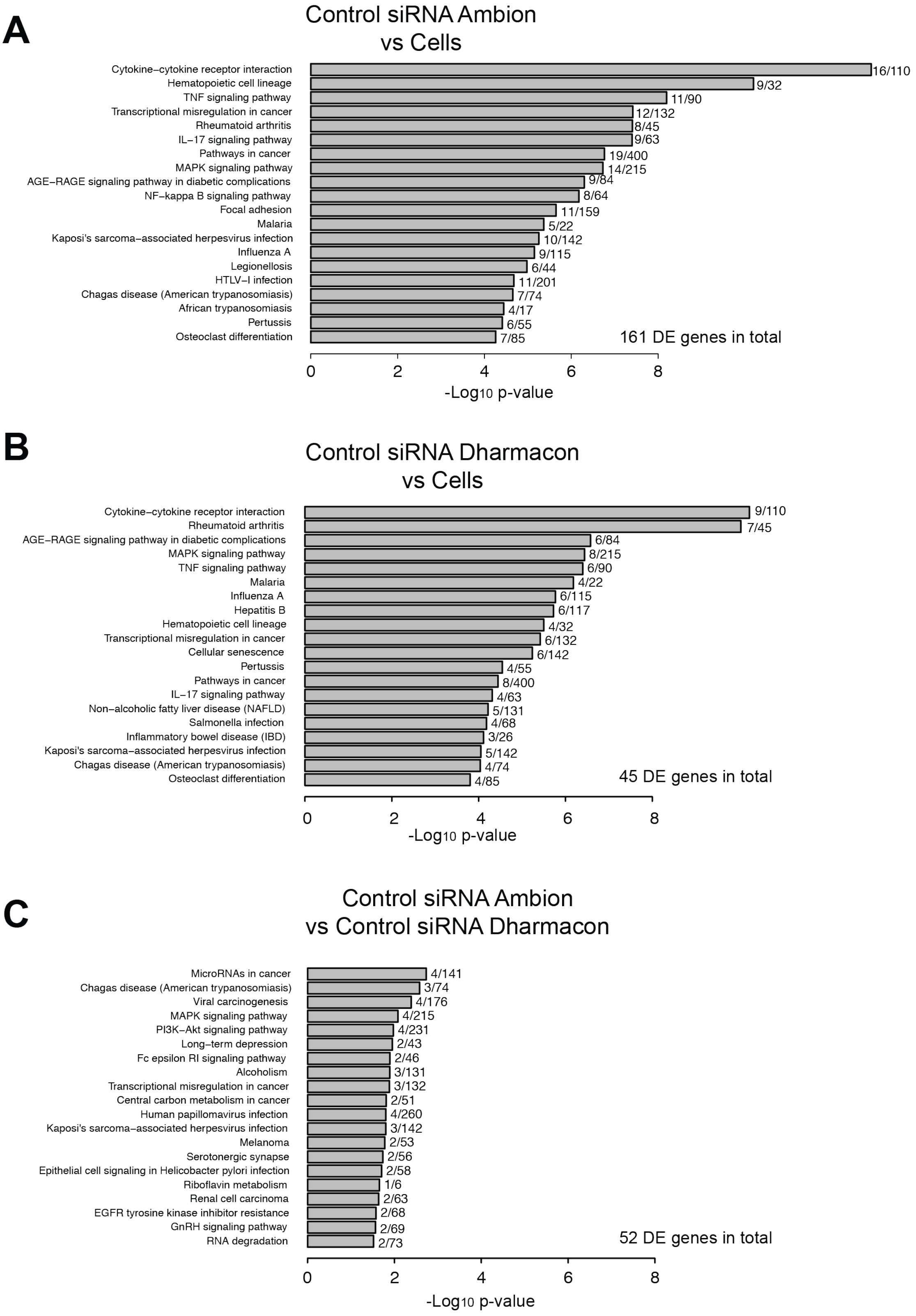
A
Control siRNA Ambion vs Cells
Cytokine−cytokine receptor interaction 16/110
Hematopoietic cell lineage 9/32
TNF signaling pathway 11/90
Transcriptional misregulation in cancer 12/132
Rheumatoid arthritis 8/45
IL−17 signaling pathway 9/63
Pathways in cancer 19/400
MAPK signaling pathway 14/215
AGE−RAGE signaling pathway in diabetic complications 9/84
NF−kappa B signaling pathway 8/64
Focal adhesion 11/159
Malaria 5/22
Kaposi's sarcoma−associated herpesvirus infection 10/142
Influenza A 9/115
Legionellosis 6/44
HTLV−I infection 11/201
Chagas disease (American trypanosomiasis) 7/74
African trypanosomiasis 4/17
Pertussis 6/55
Osteoclast differentiation 7/85
161 DE genes in total
0 2 4 6 8
-Log10 p-value
B
Control siRNA Dharmacon vs Cells
Cytokine−cytokine receptor interaction 9/110
Rheumatoid arthritis 7/45
AGE−RAGE signaling pathway in diabetic complications 6/84
MAPK signaling pathway 8/215
TNF signaling pathway 6/90
Malaria 4/22
Influenza A 6/115
Hepatitis B 6/117
Hematopoietic cell lineage 4/32
Transcriptional misregulation in cancer 6/132
Cellular senescence 6/142
Pertussis 4/55
Pathways in cancer 8/400
IL−17 signaling pathway 4/63
Non−alcoholic fatty liver disease (NAFLD) 5/131
Salmonella infection 4/68
Inflammatory bowel disease (IBD) 3/26
Kaposi's sarcoma−associated herpesvirus infection 5/142
Chagas disease (American trypanosomiasis) 4/74
Osteoclast differentiation 4/85
45 DE genes in total
0 2 4 6 8
-Log10 p-value
C
Control siRNA Ambion vs Control siRNA Dharmacon
MicroRNAs in cancer 4/141
Chagas disease (American trypanosomiasis) 3/74
Viral carcinogenesis 4/176
MAPK signaling pathway 4/215
PI3K−Akt signaling pathway 4/231
Long−term depression 2/43
Fc epsilon RI signaling pathway 2/46
Alcoholism 3/131
Transcriptional misregulation in cancer 3/132
Central carbon metabolism in cancer 2/51
Human papillomavirus infection 4/260
Kaposi's sarcoma−associated herpesvirus infection 3/142
Melanoma 2/53
Serotonergic synapse 2/56
Epithelial cell signaling in Helicobacter pylori infection 2/58
Riboflavin metabolism 1/6
Renal cell carcinoma 2/63
EGFR tyrosine kinase inhibitor resistance 2/68
GnRH signaling pathway 2/69
RNA degradation 2/73
52 DE genes in total
0 2 4 6 8
-Log10 p-value

Supplementary Figure 5. KEGG pathway analysis of DEGs from comparisons between negative control groups in the RNAi approach.

A. Top 20 KEGG terms that were most significantly overrepresented in the set of DEGs from the comparison between Ambion-treated and untreated cells.

B. Top 20 KEGG terms that were overrepresented in the set of DEGs from the comparison between GE Dharmacon (Dharm)-treated and untreated cells.

C. Top 20 KEGG terms that were overrepresented in the set of DEGs from the comparison between GE Dharmacon and Ambion-treated cells.

DEGs for each pairwise comparison were the same as those defined in Figure 1.

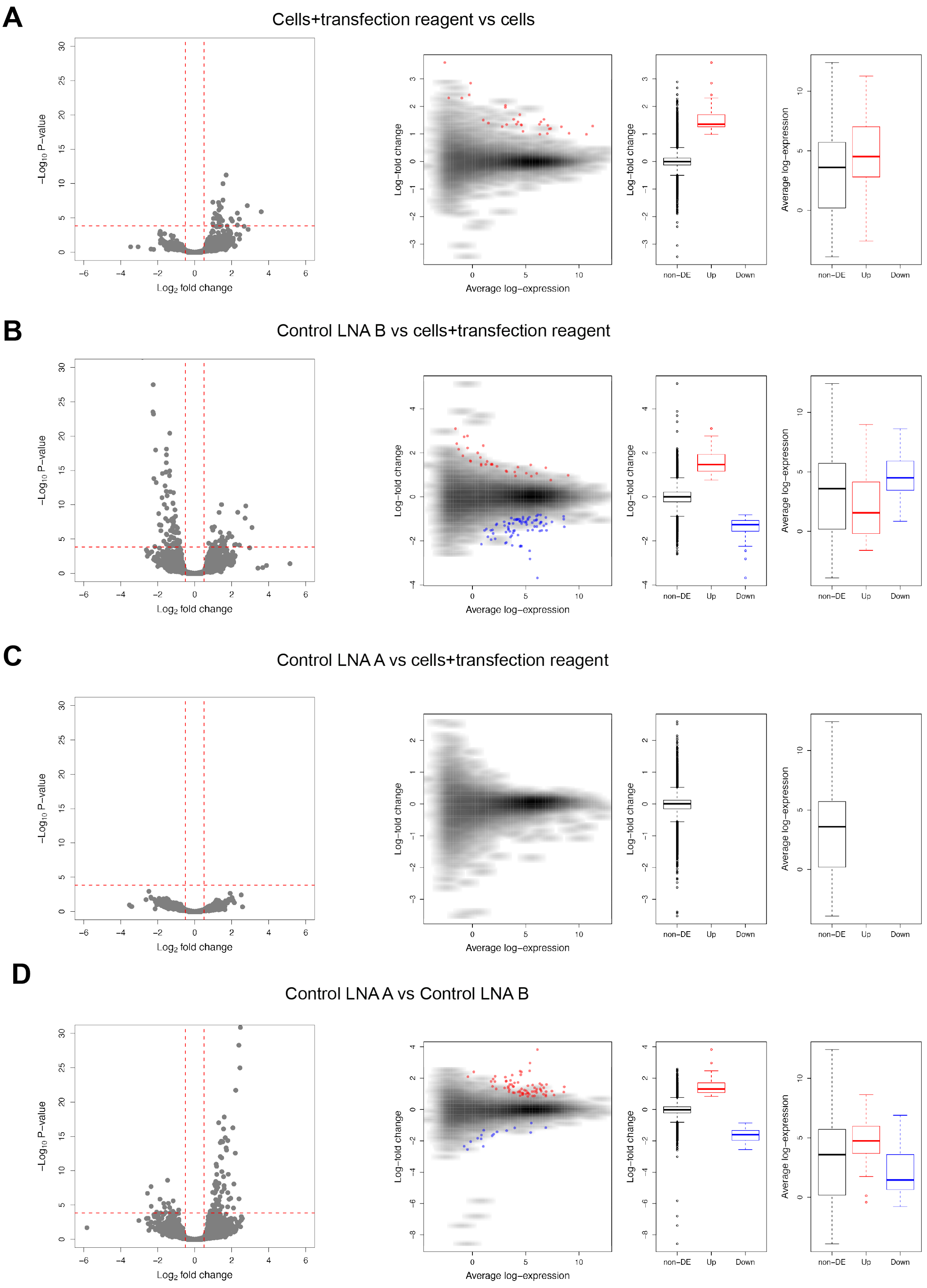

Supplementary Figure 6. Statistics of DEGs between LNA negative controls.

A volcano plot (left panel) and MA plot (centre panel) is shown for each pairwise comparison in Figure 1B. DEGs were detected at a FDR of 5% (corresponding to the dashed horizontal line in each volcano plot) after testing against a log-fold change threshold of 0.5 (dashed vertical lines). For the MA plot, the intensity of colour is proportional to the density of non-significant genes, while DEGs are highlighted as separate points. The distribution of log-fold

changes and average abundances for all DEGs changing in each direction are shown in the boxplots (right panels), along with the distributions for non-significant genes.

A. Comparison between cells treated with transfection reagent and untreated cells.

B. Comparison between cells treated with LNA negative control B and cells treated with transfection reagent.

C. Comparison between cells treated with LNA negative control A and cells treated with transfection reagent.

D. Comparison between cells treated with LNA negative control A and cells treated with LNA negative control B.

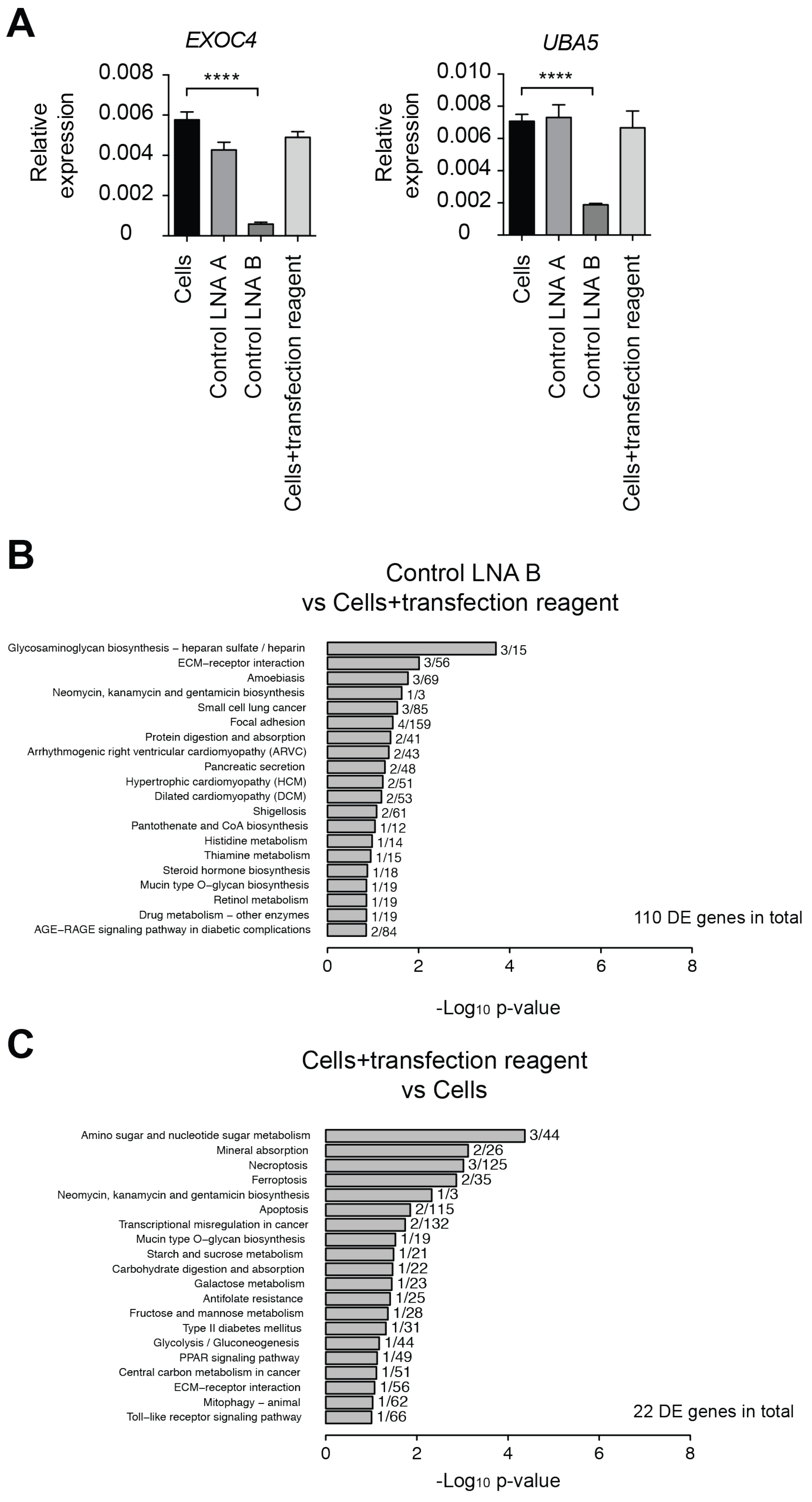
A
EXOC4
0.008
0.006
0.004
0.002
0
Relative expression
****
Cells
Control LNA A
Control LNA B
Cells+transfection reagent
UBA5
0.010
0.008
0.006
0.004
0.002
0
Relative expression
****
Cells
Control LNA A
Control LNA B
Cells+transfection reagent
B
Control LNA B vs Cells+transfection reagent
Glycosaminoglycan biosynthesis – heparan sulfate / heparin 3/15
ECM–receptor interaction 3/56
Amoebiasis 3/69
Neomycin, kanamycin and gentamicin biosynthesis 1/3
Small cell lung cancer 3/85
Focal adhesion 4/159
Protein digestion and absorption 2/41
Arrhythmogenic right ventricular cardiomyopathy (ARVC) 2/43
Pancreatic secretion 2/48
Hypertrophic cardiomyopathy (HCM) 2/51
Dilated cardiomyopathy (DCM) 2/53
Shigellosis 2/61
Pantothenate and CoA biosynthesis 1/12
Histidine metabolism 1/14
Thiamine metabolism 1/15
Steroid hormone biosynthesis 1/18
Mucin type O–glycan biosynthesis 1/19
Retinol metabolism 1/19
Drug metabolism – other enzymes 1/19
AGE–RAGE signaling pathway in diabetic complications 2/84
110 DE genes in total
0
2
4
6
8
-Log10 p-value
C
Cells+transfection reagent vs Cells
Amino sugar and nucleotide sugar metabolism 3/44
Mineral absorption 2/26
Necroptosis 3/125
Ferroptosis 2/35
Neomycin, kanamycin and gentamicin biosynthesis 1/3
Apoptosis 2/115
Transcriptional misregulation in cancer 2/132
Mucin type O–glycan biosynthesis 1/19
Starch and sucrose metabolism 1/21
Carbohydrate digestion and absorption 1/22
Galactose metabolism 1/23
Antifolate resistance 1/25
Fructose and mannose metabolism 1/28
Type II diabetes mellitus 1/31
Glycolysis / Gluconeogenesis 1/44
PPAR signaling pathway 1/49
Central carbon metabolism in cancer 1/51
ECM–receptor interaction 1/56
Mitophagy – animal 1/62
Toll–like receptor signaling pathway 1/66
22 DE genes in total
0
2
4
6
8
-Log10 p-value

Supplementary Figure 7. Validation of off-target changes in gene expression in the LNA approach using negative control oligonucleotides.

A. Expression analysis of genes (*EXOC4*, *UBA5*) affected by transfection of negative control LNA B compared to untreated HeLa cells. Expression levels were measured by qPCR and normalized to the geometric mean of *GAPDH* and *RPS18.* Error bars, s.e.m. (n=3 biological replicates). Statistical significance by two-tailed Student's t-test: **** P<0.0001.

B. Top 20 KEGG terms that were most significantly overrepresented in a pathway analysis of DEGs from the comparison of cells transfected with negative control LNA B against cells treated with transfection reagent.

C. Top 20 KEGG terms that were overrepresented in the set of DEGs from the comparison of cells treated with transfection reagent compared to untreated cells.

DEGs for each pairwise comparison were the same as those defined in Figure 1.

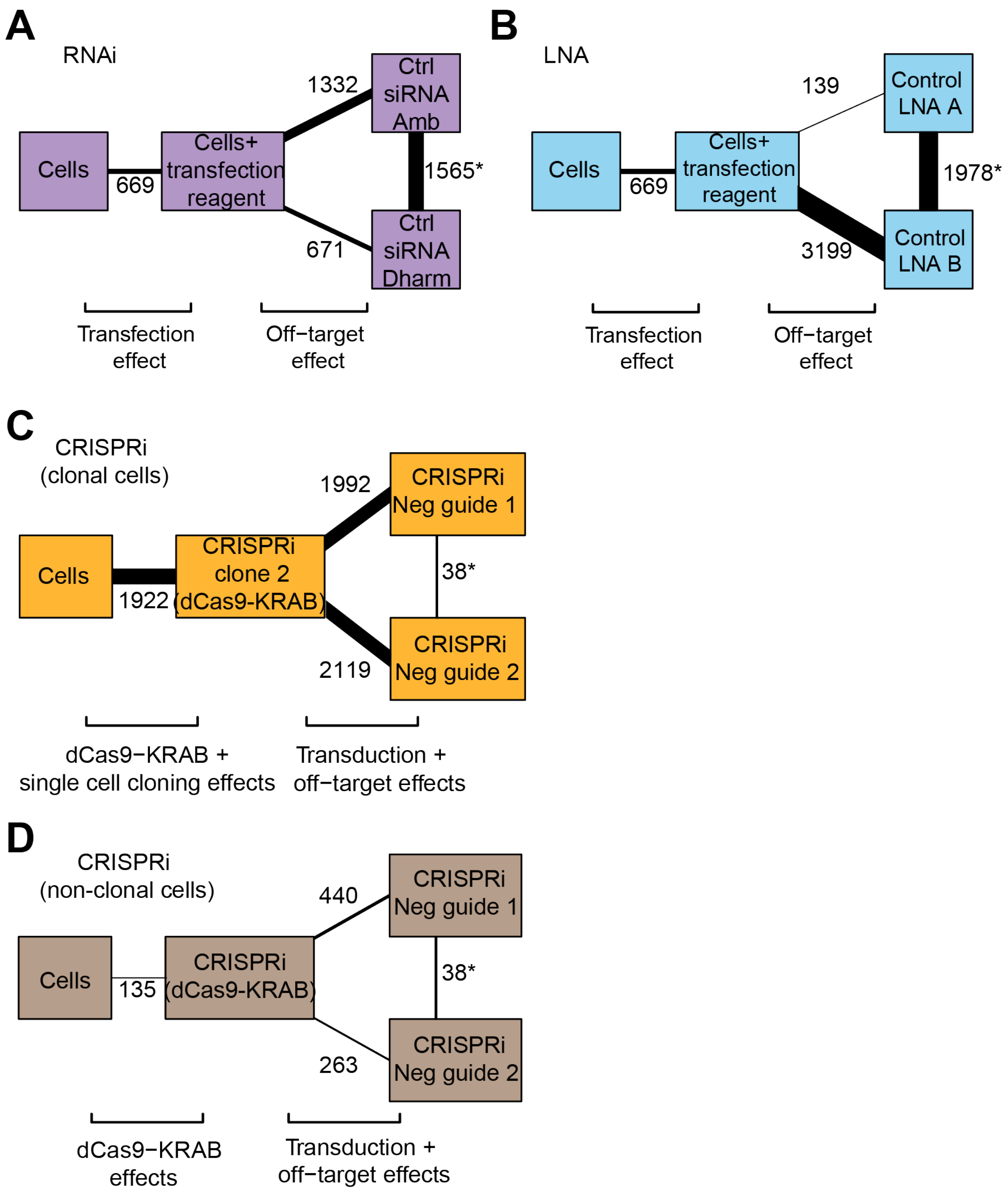

Supplementary Figure 8. Off-target effects associated with three LOF methods based on DEGs detected without any log-fold change threshold.

A. Comparison of the transcriptional differences between untreated cells and cells treated with two negative control siRNAs from Ambion (Amb) and GE Dharmacon (Dharm). The number of DEGs detected between each pair of treatments is labelled and shown as connecting lines of proportional thickness.

B. Comparison of transcriptional differences between untreated cells, cells treated with transfection reagent and two negative control LNAs (A and B). The number of genes differing between each pair of treatments is labelled as described in **A**.

C. Comparison of transcriptional differences between parental cells, clonal cells expressing

only dCas9-KRAB (CRISPRi clone 2) and clonal cells treated with two negative control guide RNAs (negative guide 1 and 2). The number of genes differing between each pair of treatments is labelled as described in **A**.

D. Comparison of the transcriptional differences between parental cells, non-clonal cells expressing only dCas9-KRAB and non-clonal cells treated with two negative control guide RNAs (negative guide 1 and 2). The number of genes differing between each pair of treatments is labelled as described in **A**.

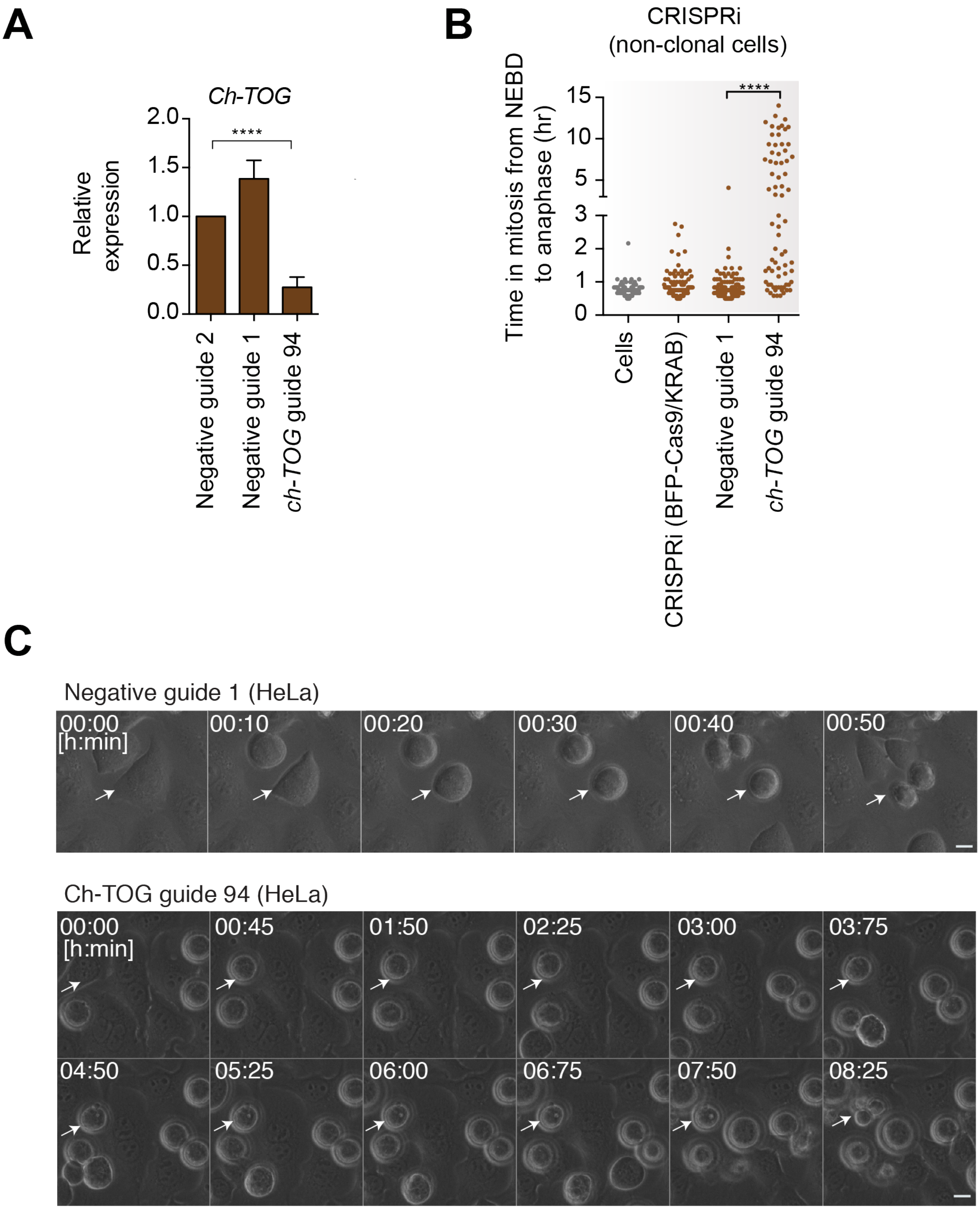

Supplementary Figure 9. Successful depletion of a protein-coding gene *Ch-TOG* using CRISPRi in non-clonal cells.

A. CRISPRi-mediated repression of *Ch-TOG* resulted in 80% reduction of *Ch-TOG* compared to negative guide RNA 2 in non-clonal HeLa cells. Expression levels were measured by qPCR and normalized to the geometric mean of *GAPDH* and *RPS18.* Error bars, s.e.m. (n=3 biological replicates). Statistical significance by two-tailed Student's t-test: **** P<0.0001.

B. Quantification results from time-lapse microscopy of mitotic progression of HeLa cells after CRISPRi-mediated depletion of *Ch-TOG* in non-clonal cells. Bars show mean

(*n*=one biological replicate). Statistical significance by Mann-Whitney test: ****P<0.0001. NEBD=nuclear envelope breakdown.

C. Representative still images from the time-lapse microscopy. White arrows depict cell going through mitosis. Scale bar indicates 20μm.

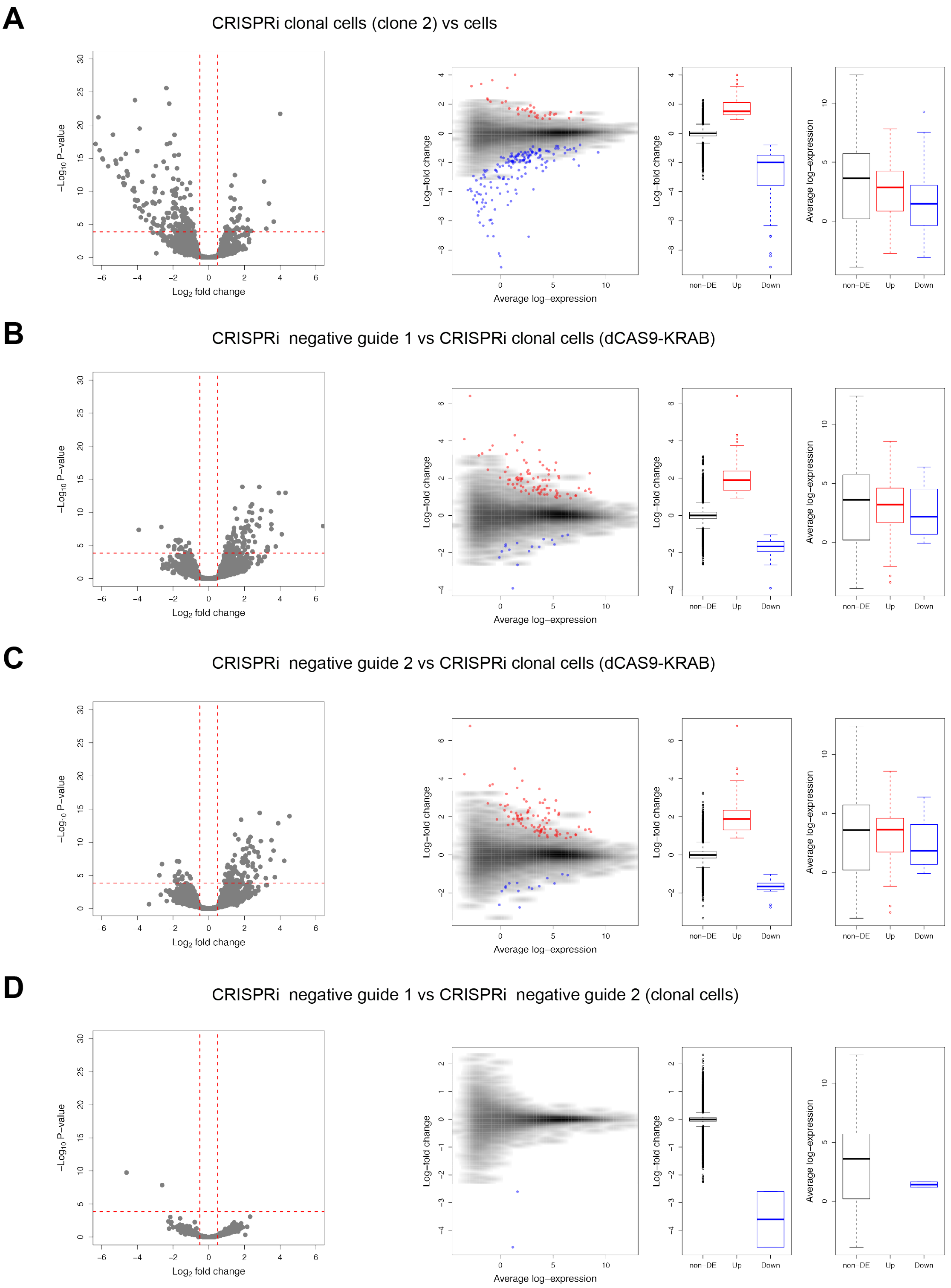

Supplementary Figure 10. Statistics of DEGs between CRISPRi negative controls in clonal cells.

A volcano plot (left panel) and MA plot (centre panel) is shown for each pairwise comparison in Figure 2B. DEGs were detected at a FDR of 5% (corresponding to the dashed horizontal line in each volcano plot) after testing against a log-fold change threshold of 0.5 (dashed vertical lines). For the MA plot, the intensity of colour is proportional to the density of non-significant genes, while DEGs are highlighted as separate points. The distribution of log-fold

changes and average abundances for all DEGs changing in each direction are shown in the boxplots (right panels), along with the distributions for non-significant genes.

A. Comparison between CRISPRi clonal cells (clone 2) and untreated cells.

B. Comparison between CRISPRi clonal cells transduced with negative guide 1 and CRISPRi clonal cells.

C. Comparison between CRISPRi clonal cells transduced with negative guide 2 and CRISPRi clonal cells

D. Comparison between CRISPRi clonal cells transduced with negative guide 1 and CRISPRi clonal cells transduced with negative guide 2.

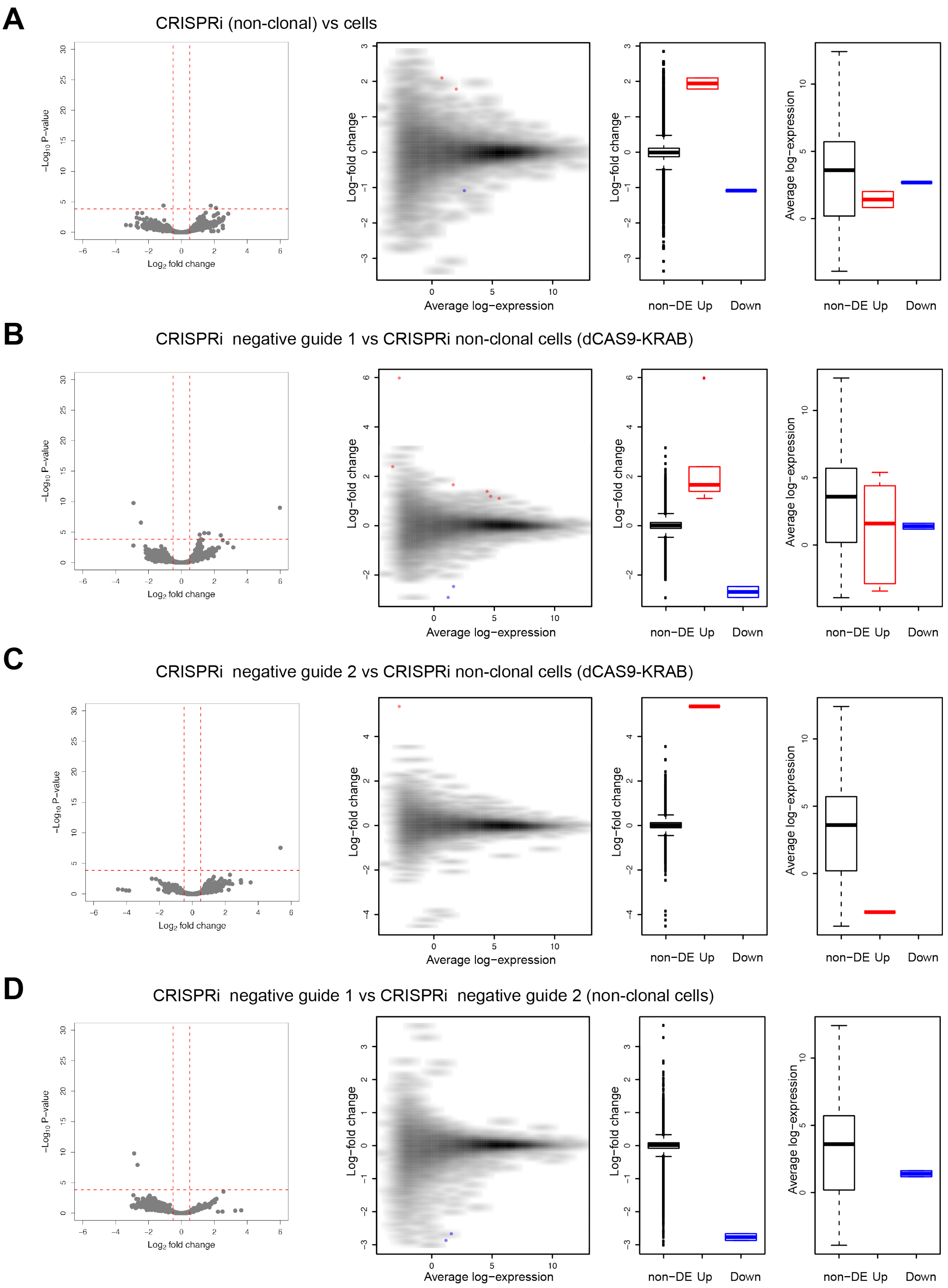

Supplementary Figure 11. Statistics for DEGs between CRISPRi negative controls in non-clonal cells.

A volcano plot (left panel) and MA plot (centre panel) is shown for each pairwise comparison in Figure 2C. DEGs were detected at a FDR of 5% (corresponding to the dashed horizontal line in each volcano plot) after testing against a log-fold change threshold of 0.5 (dashed vertical lines). For the MA plot, the intensity of colour is proportional to the density of non-significant genes, while DEGs are highlighted as separate points. The distribution of log-fold

changes and average abundances for all DEGs changing in each direction are shown in the boxplots (right panels), along with the distributions for non-significant genes.

A. Comparison between CRISPRi non-clonal cells cells and untreated cells.

B. Comparison between CRISPRi non-clonal cells transduced with negative guide 1 and CRISPRi non-clonal cells.

C. Comparison between CRISPRi non-clonal cells transduced with negative guide 2 and CRISPRi non-clonal cells

D. Comparison between CRISPRi non-clonal cells transduced with negative guide 1 and CRISPRi non-clonal cells transduced with negative guide 2.

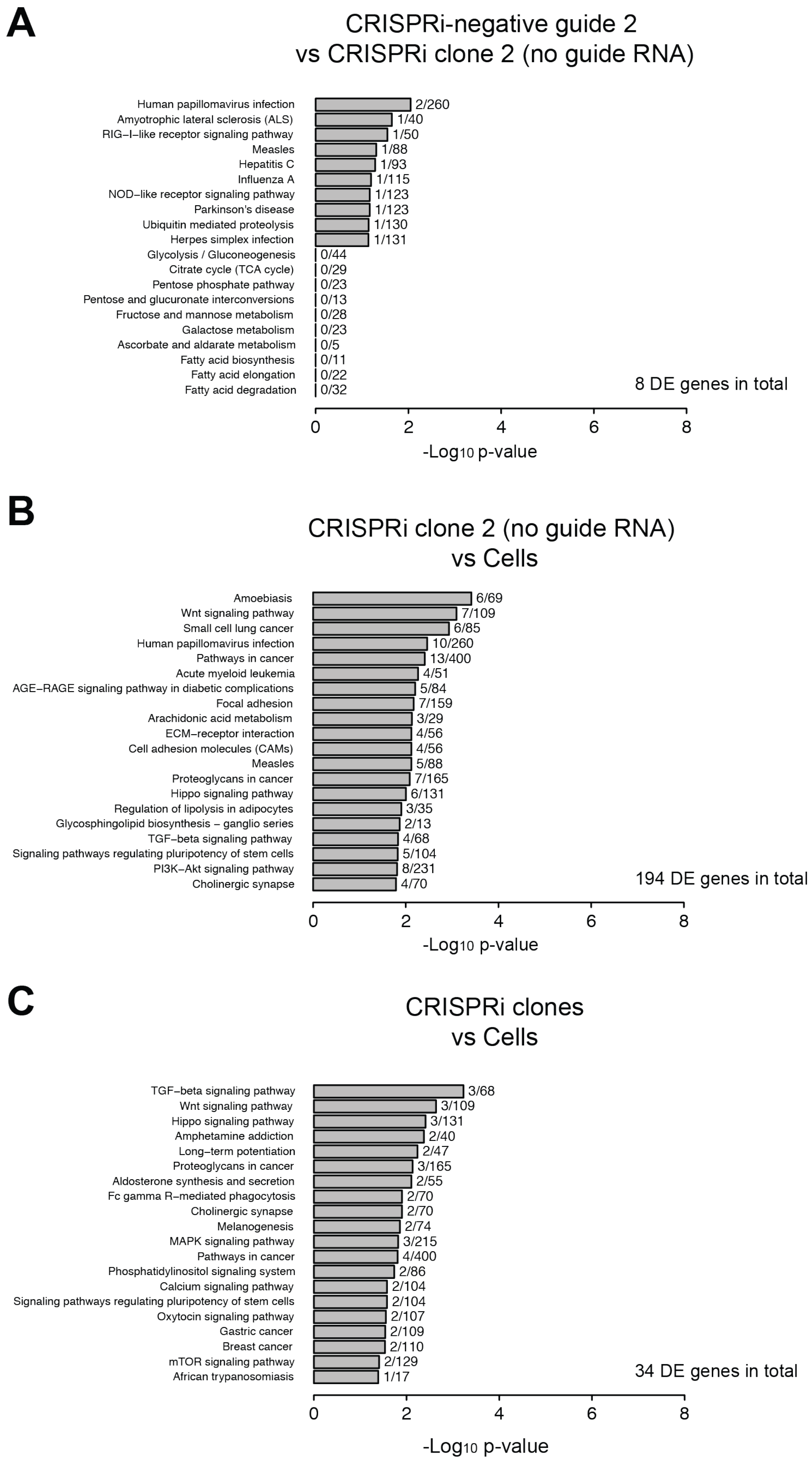
A
CRISPRi-negative guide 2
vs CRISPRi clone 2 (no guide RNA)
Human papillomavirus infection 2/260
Amyotrophic lateral sclerosis (ALS) 1/40
RIG−I−like receptor signaling pathway 1/50
Measles 1/88
Hepatitis C 1/93
Influenza A 1/115
NOD−like receptor signaling pathway 1/123
Parkinson's disease 1/123
Ubiquitin mediated proteolysis 1/130
Herpes simplex infection 1/131
Glycolysis / Gluconeogenesis 0/44
Citrate cycle (TCA cycle) 0/29
Pentose phosphate pathway 0/23
Pentose and glucuronate interconversions 0/13
Fructose and mannose metabolism 0/28
Galactose metabolism 0/23
Ascorbate and aldarate metabolism 0/5
Fatty acid biosynthesis 0/11
Fatty acid elongation 0/22
Fatty acid degradation 0/32
8 DE genes in total
0 2 4 6 8
-Log10 p-value
B
CRISPRi clone 2 (no guide RNA)
vs Cells
Amoebiasis 6/69
Wnt signaling pathway 7/109
Small cell lung cancer 6/85
Human papillomavirus infection 10/260
Pathways in cancer 13/400
Acute myeloid leukemia 4/51
AGE−RAGE signaling pathway in diabetic complications 5/84
Focal adhesion 7/159
Arachidonic acid metabolism 3/29
ECM−receptor interaction 4/56
Cell adhesion molecules (CAMs) 4/56
Measles 5/88
Proteoglycans in cancer 7/165
Hippo signaling pathway 6/131
Regulation of lipolysis in adipocytes 3/35
Glycosphingolipid biosynthesis − ganglio series 2/13
TGF−beta signaling pathway 4/68
Signaling pathways regulating pluripotency of stem cells 5/104
PI3K−Akt signaling pathway 8/231
Cholinergic synapse 4/70
194 DE genes in total
0 2 4 6 8
-Log10 p-value
C
CRISPRi clones
vs Cells
TGF−beta signaling pathway 3/68
Wnt signaling pathway 3/109
Hippo signaling pathway 3/131
Amphetamine addiction 2/40
Long−term potentiation 2/47
Proteoglycans in cancer 3/165
Aldosterone synthesis and secretion 2/55
Fc gamma R−mediated phagocytosis 2/70
Cholinergic synapse 2/70
Melanogenesis 2/74
MAPK signaling pathway 3/215
Pathways in cancer 4/400
Phosphatidylinositol signaling system 2/86
Calcium signaling pathway 2/104
Signaling pathways regulating pluripotency of stem cells 2/104
Oxytocin signaling pathway 2/107
Gastric cancer 2/109
Breast cancer 2/110
mTOR signaling pathway 2/129
African trypanosomiasis 1/17
34 DE genes in total
0 2 4 6 8
-Log10 p-value

Supplementary Figure 12. KEGG pathway analysis of DEGs from comparisons between negative control groups in CRISPRi-based approaches.

A. Top 20 KEGG terms that were most significantly overrepresented in a pathway analysis of DEGs from the comparison between clone 2 without any guide RNA and clone 2 after transduction with negative guide RNA 2.

B. Top 20 KEGG terms overrepresented in the set of DEGs from the comparison between clone 2 in the absence of any guide RNA and untransduced HeLa cells.

C Top 20 KEGG terms overrepresented in the set of 37 genes that were differentially expressed in all three clones compared to untransduced HeLa cells.

DEGs for each pairwise comparison were the same as those defined in Figure 2.

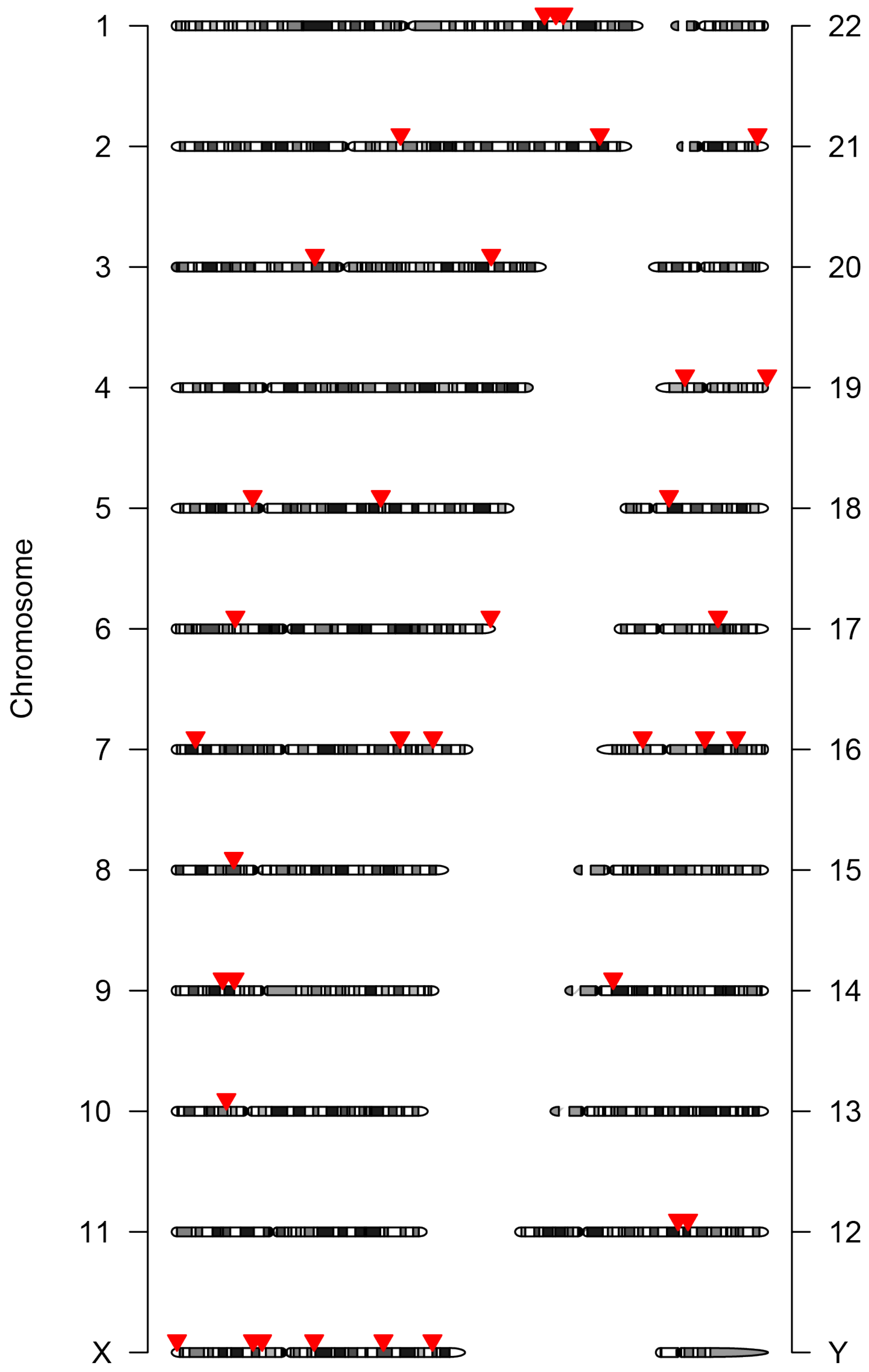

Supplementary Figure 13. Chromosomal locations of the 37 genes identified as a common transcriptomic signature of single-cell cloning in CRISPRi. This plot was generated using the prepareGenomePlot from the quantsmooth package (https://bioconductor.org/packages/quantsmooth) using the midpoint of each gene body.

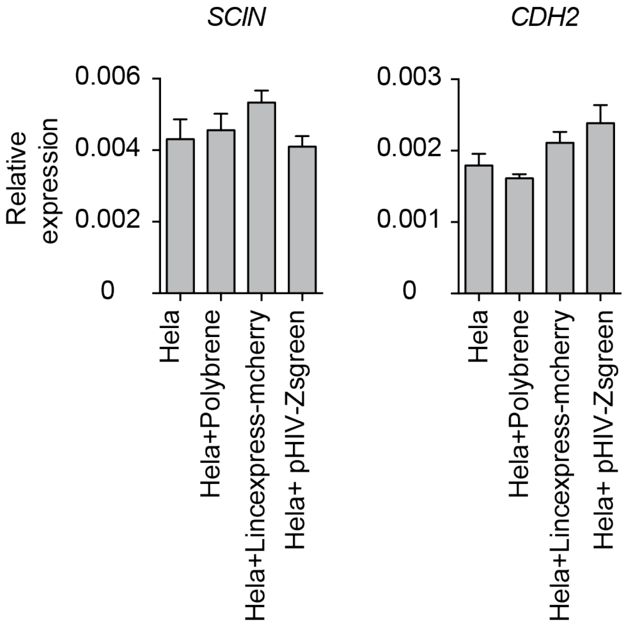

Supplementary Figure 14. Lentiviral transduction is not responsible for the differential expression of genes that are detected after dCas9-KRAB transduction in the CRISPRi approach. Expression of *SCIN* and *CDH2* was not affected by transduction of cells either with polybrene or with two different lentiviral vectors (LincExpress-mcherry and pHIV-Zsgreen) compared to HeLa untreated cells. For all graphs, expression levels were measured by qPCR and normalized to the geometric mean of *GAPDH* and *RPS18.* Error bars, s.e.m. (*n*=3 biological replicates).

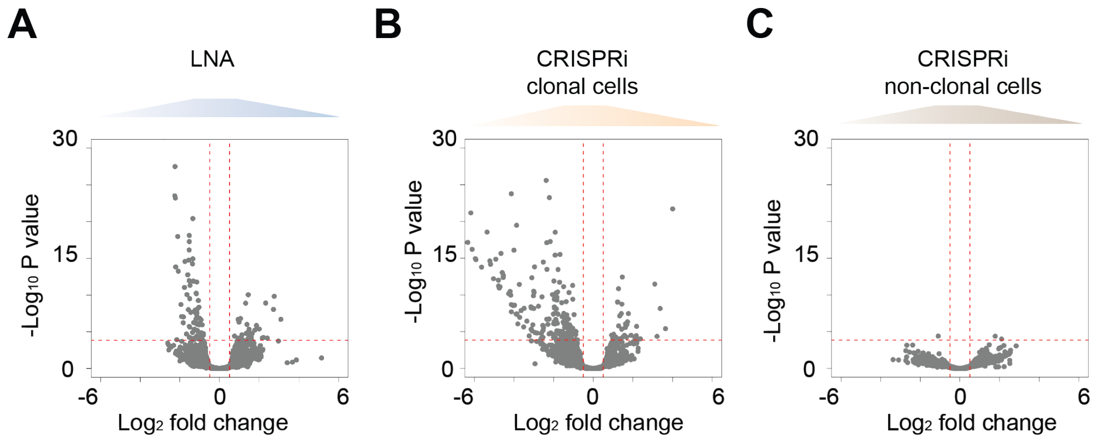

Supplementary Figure 15. The number and effect size of DEGs is greater dCas9-KRAB transduction in CRISPRi clonal cells compared to non-clonal cells or cells transfected with negative control oligonucleotides in the LNA approach.

A. Volcano plot of 112 DEGs (see Figure 1B) between cells treated with negative control LNA B and cells treated with transfection reagent.

B. Volcano plot of 201 DEGs (see Figure 2B) between untransduced cells and CRISPRi clonal cells in the absence of any guide RNA.

C. Volcano plot of three DEGs (see Figure 2C) between untransduced cells and CRISPRi non-clonal cells in the absence of any guide RNA.

The red horizontal line represents the significance threshold corresponding to an FDR of 5%. Red vertical lines represent $\log_2$-fold change thresholds of +/- 0.5, while the black vertical lines represent $\log_2$-fold change thresholds of +/- 3.

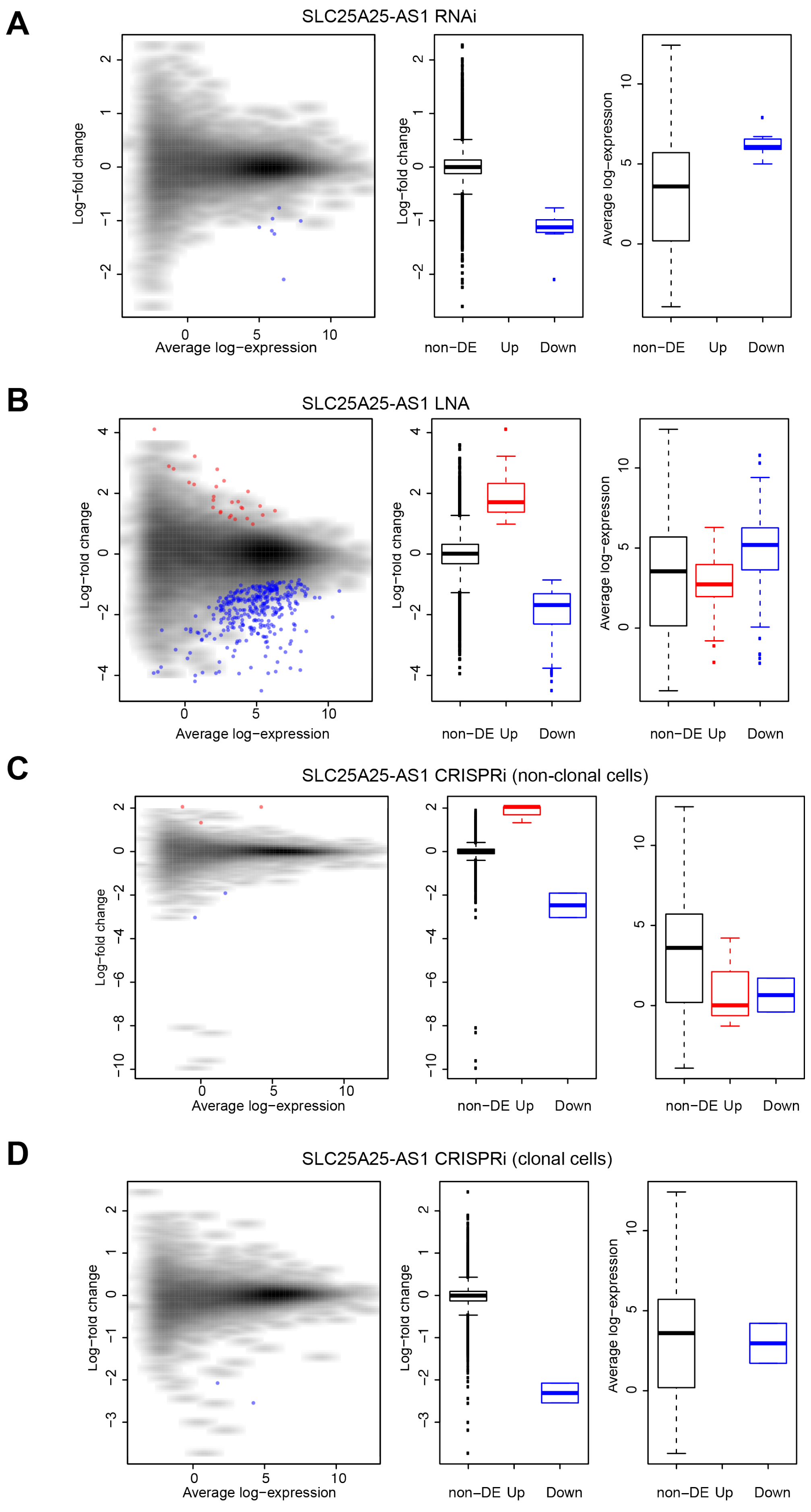
A
SLC25A25-AS1 RNAi
Log−fold change
Average log−expression
non−DE
Up
Down
Average log−expression
B
SLC25A25-AS1 LNA
Log−fold change
Average log−expression
non−DE
Up
Down
Average log−expression
C
SLC25A25-AS1 CRISPRi (non-clonal cells)
Log−fold change
Average log−expression
non−DE
Up
Down
D
SLC25A25-AS1 CRISPRi (clonal cells)
Log−fold change
Average log−expression
non−DE
Up
Down
Average log−expression

Supplementary Figure 16. Statistics of DEGs upon depletion of *SLC25A25-AS1* with each LOF method.

A MA plot (left panel) is shown for the effect of depletion with each LOF method. DEGs were defined in the same manner as in the volcano plots in Figure 4B. The intensity of colour in each MA plot is proportional to the density of non-significant genes, while DEGs are highlighted as separate points. The distribution of log-fold changes and average abundances for all DEGs changing in each direction are shown in the boxplots (right panels), along with the distributions for non-significant genes.

A. Comparison between *SLC25A25-AS1* RNAi treated cells and cells treated with negative control siRNAs.

B. Comparison between *SLC25A25-AS1* LNA 2 treated cells and cells treated with negative control LNA oligonucleotides.

C. Comparison between *SLC25A25-AS1* CRISPRi treated cells and cells transduced with negative guide 2 (CRISPRi non-clonal cells).

D. Comparison between *SLC25A25-AS1* CRISPRi treated cells and cells transduced with negative guide 2 (CRISPRi clonal cells).

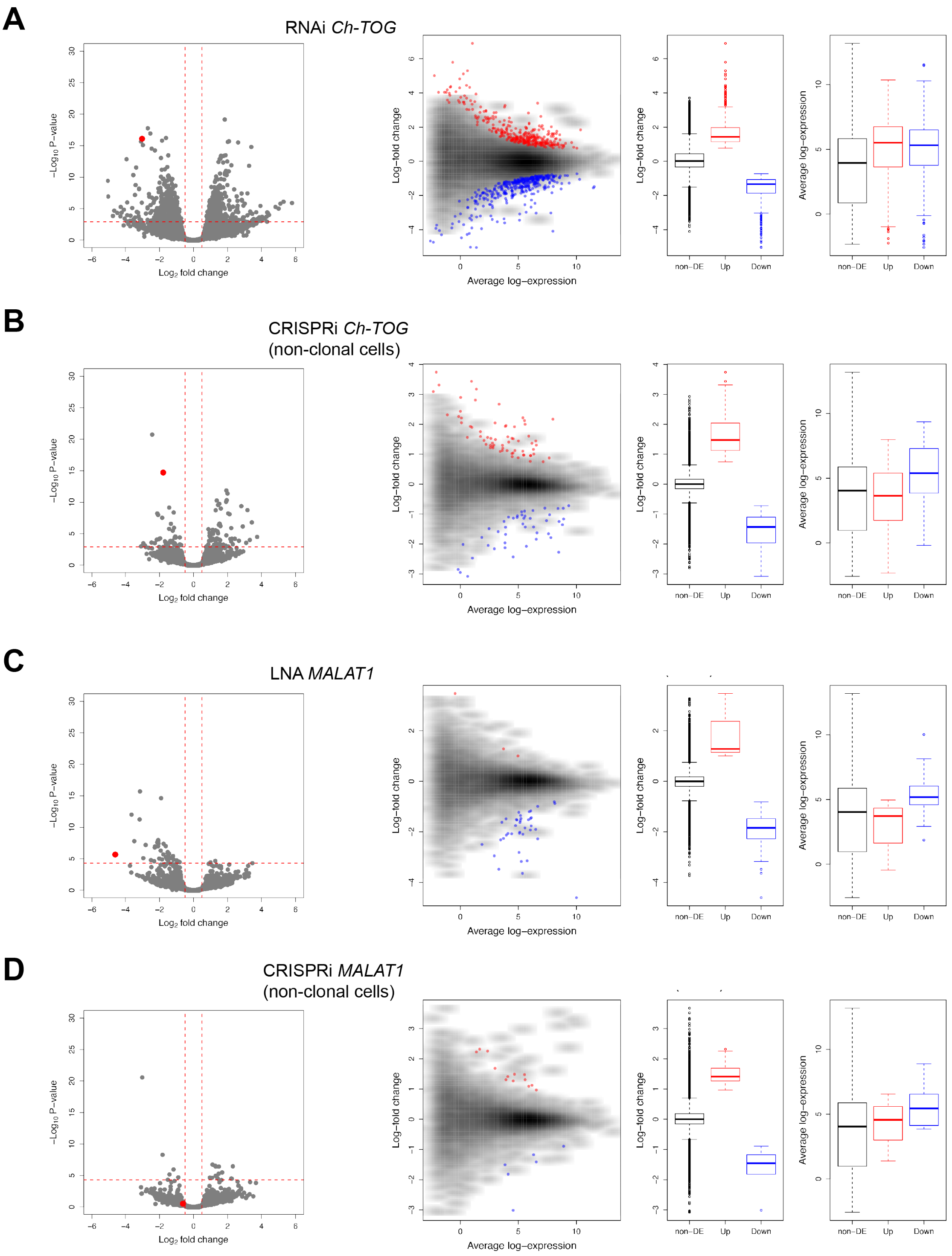

Supplementary Figure 17. Statistics of DEGs upon depletion of *MALAT1* or *Ch-TOG* with a variety of LOF methods.

A volcano plot (left panel) and a MA plot (centre plot) is shown for each pairwise comparison in Figure 5. DEGs were detected at a FDR of 5% (corresponding to the dashed horizontal line in each volcano plot) after testing against a log-fold change threshold of 0.5 (dashed vertical lines). For the MA plot, the intensity of colour is proportional to the density of non-significant genes, while DEGs are highlighted as separate points. The distribution of log-fold changes

and average abundances for all DEGs changing in each direction are shown in the boxplots (right panels), along with the distributions for non-significant genes.

A. Comparison between *Ch-TOG* RNAi treated cells and cells treated with negative control siRNA (GE Dharmacon).

B. Comparison between *Ch-TOG* CRISPRi treated cells and cells transduced with negative guide 2 (CRISPRi non-clonal cells).

C. Comparison between *MALAT1* LNA treated cellls and cells transfected with negative control LNA A.

D. Comparison between *MALAT1* CRISPRi treated cells and cells transduced with negative guide 2 (CRISPRi non-clonal cells).

The red dot in the volcano plot corresponds to the depleted gene of interest.

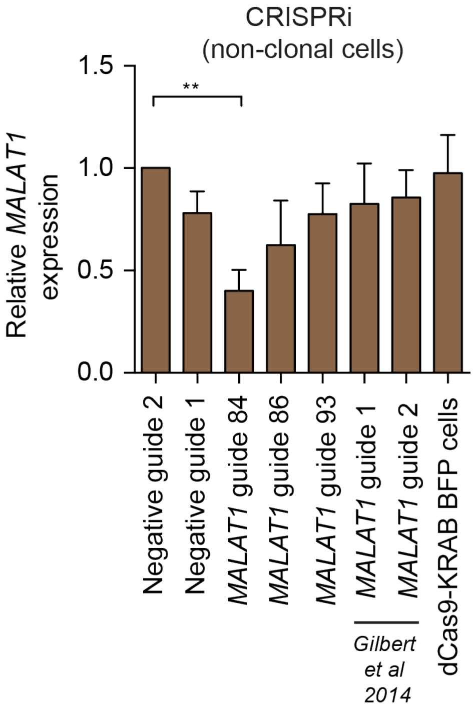

Supplementary Figure 18. Depletion of *MALAT1* using the CRISPRi method.
CRISPRi-mediated repression of *MALAT1* in non-clonal cells relative to the negative guide RNA 2 was analysed by qPCR 48 hr post-transduction. Five different guides targeting different regions of *MALAT1* were tested but only guide 84 yielded 50% reduction in *MALAT1* level. Expression levels of *MALAT1* was normalized to the geometric mean of *GAPDH* and *RPS18.* Error bars, s.e.m. (*n*=3 biological replicates for guides 84, 86 and 93; *n*=2 biological replicates for guides 1 and 2). Statistical significance by two-tailed Student's *t*-test: ** P<0.01.

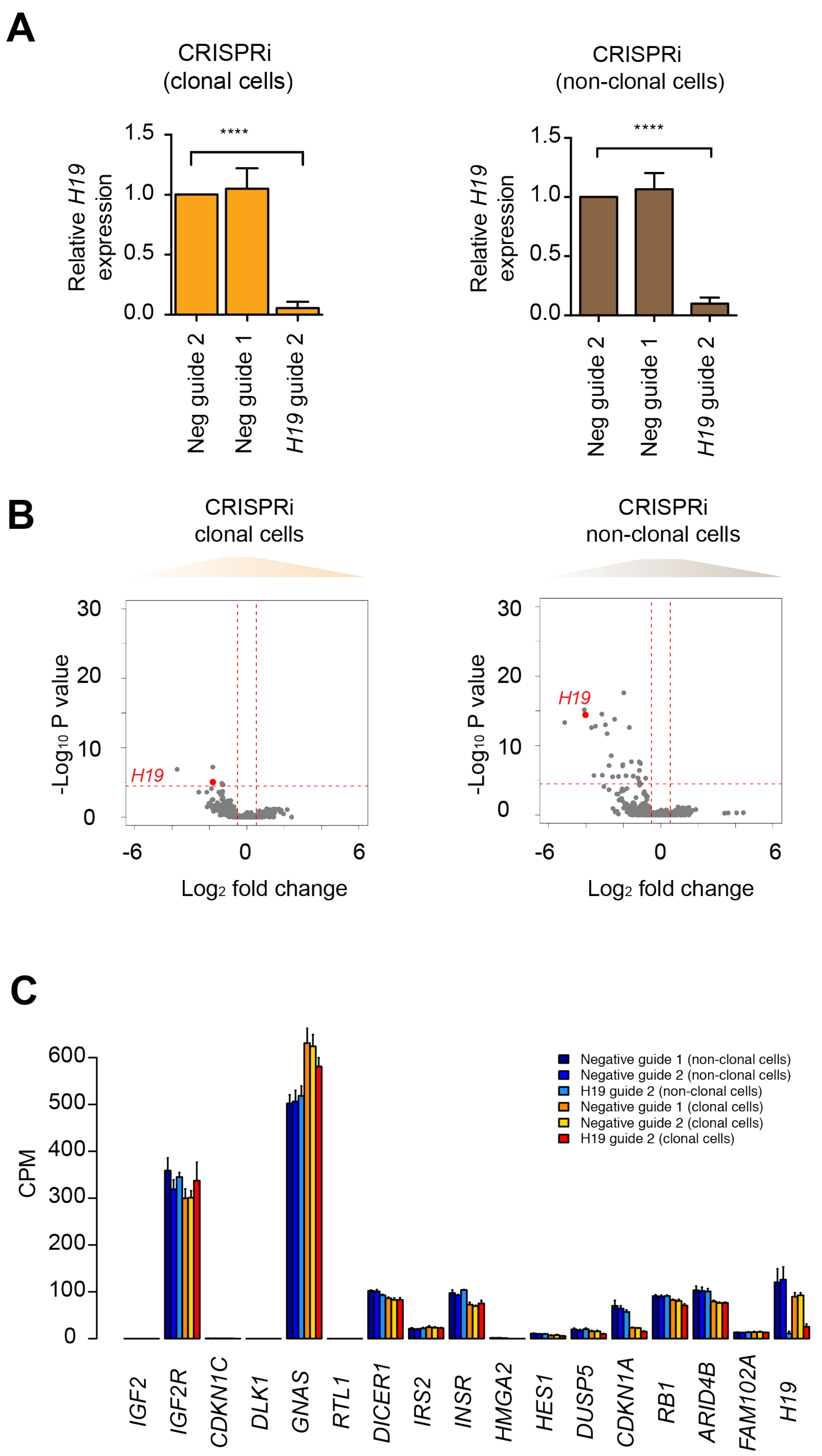
A
CRISPRi
(clonal cells)
Relative H19 expression
1.5
1.0
0.5
0.0
****
Neg guide 2
Neg guide 1
H19 guide 2
CRISPRi
(non-clonal cells)
Relative H19 expression
1.5
1.0
0.5
0.0
****
Neg guide 2
Neg guide 1
H19 guide 2
B
CRISPRi
clonal cells
-Log10 P value
30
20
10
0
H19
-6
0
6
Log2 fold change
CRISPRi
non-clonal cells
-Log10 P value
30
20
10
0
H19
-6
0
6
Log2 fold change
C
CPM
600
500
400
300
200
100
0
Negative guide 1 (non-clonal cells)
Negative guide 2 (non-clonal cells)
H19 guide 2 (non-clonal cells)
Negative guide 1 (clonal cells)
Negative guide 2 (clonal cells)
H19 guide 2 (clonal cells)
IGF2
IGF2R
CDKN1C
DLK1
GNAS
RTL1
DICER1
IRS2
INSR
HMGA2
HES1
DUSP5
CDKN1A
RB1
ARID4B
FAM102A
H19

Supplementary Figure 19. Successful depletion of *H19* using the CRISPRi method.

A. CRISPRi-mediated repression of *H19* in clonal (left panel) and non-clonal cells (right panel) relative to the negative guide RNA 2 was analysed by qPCR. Expression level of *H19* was normalized to the geometric mean of *GAPDH* and *RPS18.* Error bars, s.e.m. (*n*=4 biological replicates). Statistical significance by two-tailed Student's *t*-test: **** P<0.0001.

B. Volcano plots of transcriptional differences induced by CRISPRi-mediated depletion of *H19* in clonal (left panel) and non-clonal CRISPRi cells (right panel) compared to two negative guide RNAs (1 and 2). 5 and 29 DEGs were detected in clonal and non-clonal cells, respectively (Supplementary Table 3).

C. Expression in counts-per-million (CPM) of downstream targets of *H19* after CRISRPi-mediated depletion in clonal and non-clonal cells and RNA-seq.

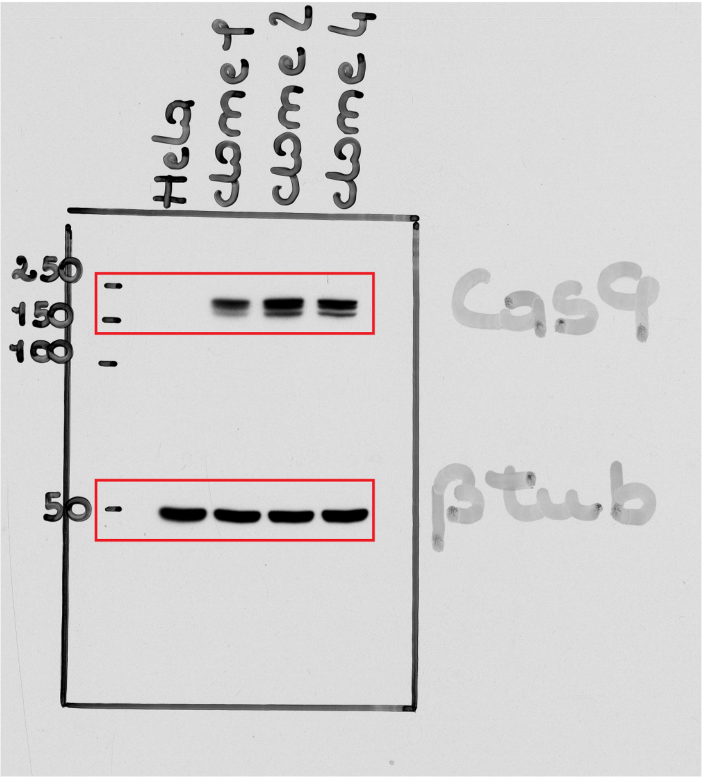

Supplementary Figure 20. An uncropped picture of the immunoblot of dCas9-KRAB in parental cells (HeLa) and each CRISPRi clone.

Regions in red boxes are shown in Figure 2. CRISPRi clone 4 is renamed to CRISPRi clone 3 in the text.

Supplementary references

1. Liao, Y., Smyth, G.K. & Shi, W. The Subread aligner: fast, accurate and scalable read mapping by seed-and-vote. *Nucleic Acids Res* **41**, e108 (2013).
2. Liao, Y., Smyth, G.K. & Shi, W. featureCounts: an efficient general purpose program for assigning sequence reads to genomic features. *Bioinformatics* **30**, 923-930 (2014).
3. Ritchie, M.E. et al. limma powers differential expression analyses for RNA-sequencing and microarray studies. *Nucleic Acids Res* **43**, e47 (2015).
4. Robinson, M.D. & Oshlack, A. A scaling normalization method for differential expression analysis of RNA-seq data. *Genome Biol* **11**, R25 (2010).
5. Law, C.W., Chen, Y., Shi, W. & Smyth, G.K. voom: Precision weights unlock linear model analysis tools for RNA-seq read counts. *Genome Biol* **15**, R29 (2014).
6. Phipson, B., Lee, S., Majewski, I.J., Alexander, W.S. & Smyth, G.K. Robust hyperparameter estimation protects against hypervariable genes and improves power to detect differential expression. *Annals of Applied Statistics* **10**, 946-963 (2016).
7. McCarthy, D.J. & Smyth, G.K. Testing significance relative to a fold-change threshold is a TREAT. *Bioinformatics* **25**, 765-771 (2009).
8. Berger, R.L. & Hsu, J.C. Bioequivalence trials, intersection-union tests and equivalence confidence sets. *Statistical Science* **11**, 283-319 (1996).
9. O'Geen, H., Henry, I.M., Bhakta, M.S., Meckler, J.F. & Segal, D.J. A genome-wide analysis of Cas9 binding specificity using ChIP-seq and targeted sequence capture. *Nucleic Acids Res* **43**, 3389-3404 (2015).
10. Thakore, P.I. et al. Highly specific epigenome editing by CRISPR-Cas9 repressors for silencing of distal regulatory elements. *Nat Methods* **12**, 1143-1149 (2015).
11. Zhang, Y. et al. Model-based analysis of ChIP-Seq (MACS). *Genome Biol* **9**, R137 (2008).
12. Lawrence, M., Gentleman, R. & Carey, V. rtracklayer: an R package for interfacing with genome browsers. *Bioinformatics* **25**, 1841-1842 (2009).
